# Multifunctional multi-shank neural probe for investigating and modulating long-range neural circuits in vivo

**DOI:** 10.1038/s41467-019-11628-5

**Published:** 2019-08-22

**Authors:** Hyogeun Shin, Yoojin Son, Uikyu Chae, Jeongyeon Kim, Nakwon Choi, Hyunjoo J. Lee, Jiwan Woo, Yakdol Cho, Soo Hyun Yang, C. Justin Lee, Il-Joo Cho

**Affiliations:** 1 Center for BioMicrosystems, Brain Science Institute, Korea Institute of Science and Technology (KIST), Seoul, Korea; 2 Division of Bio-Medical Science & Technology, KIST School, Korea University of Science and Technology (UST), Daejeon, Korea; 3 School of Electrical Engineering, Korea University, Seoul, Korea; 4 Korea Brain Research Institute, Daegu, Korea; 5 School of Electrical Engineering, Korea Advanced Institute of Science and Technology (KAIST), Daejeon, Korea; 6 Center for Neuroscience, Brain Science Institute, Korea Institute of Science and Technology (KIST), Seoul, Korea; 7 Department of Anatomy, College of Medicine, Korea University, Seoul, Korea

**Keywords:** Engineering, Biomedical engineering, Biological techniques, Neural circuits

## Abstract

Investigation and modulation of neural circuits in vivo at the cellular level are very important for studying functional connectivity in a brain. Recently, neural probes with stimulation capabilities have been introduced, and they provided an opportunity for studying neural activities at a specific region in the brain using various stimuli. However, previous methods have a limitation in dissecting long-range neural circuits due to inherent limitations on their designs. Moreover, the large size of the previously reported probes induces more significant tissue damage. Herein, we present a multifunctional multi-shank MEMS neural probe that is monolithically integrated with an optical waveguide for optical stimulation, microfluidic channels for drug delivery, and microelectrode arrays for recording neural signals from different regions at the cellular level. In this work, we successfully demonstrated the functionality of our probe by confirming and modulating the functional connectivity between the hippocampal CA3 and CA1 regions in vivo.

## Abstract (web summary)

Microelectromechanical neural probes can cause tissue damage and often cannot record from distant brain areas. Here the authors combine electrical recording, optical stimulation and microfluidic drug delivery in one multi-shank probe with thinner shanks to reduce damage and a flexible design to target long-range neural circuits.

## Introduction

Investigating neural circuits in the brain is very important for understanding the fundamental mechanisms of brain functions and providing a therapeutic method for neurological disorders^1–3^. Over the past several decades, electrophysiological studies with brain slices of animals, such as mice and rats, have yielded numerous findings concerning the functional connectivities of both physiological^4–6^ and pathological^7–9^ neural circuits. However, the investigation of neural circuits with the slices has been limited to two-dimensional (2D) local circuits due to an inherent issue of the use of physically disconnected and damaged sections. Recently, advances in microelectromechanical systems (MEMS) neural probes have provided various possibilities for studying the neural activities in intact brains^10–12^. Also, various stimulation capabilities have been integrated on the MEMS neural probe, and these probes have provided the way to modulate neural circuit accurately. Notably, the cell-type-specific stimulation with optogenetics has allowed the precise modulation of neural circuits^13–15^. Besides, the delivery of neurochemicals that influence synaptic transmission has also had a pivotal role in studying neural circuits^16,17^. Several neural probes integrated with different kinds of capabilities have been introduced for more accurate control of neural circuits in vivo. However, all of the previously reported probes possess inherent limitations on their designs and thus cannot be used for constructing in an expandable array structure^18–21^, which is indispensable for examining neural circuits in an all-embracing manner within the three-dimensional (3D) space of the brain. For example, a multifunctional fibre was fabricated through thermally thinning polymeric components (e.g. an electrode material), which allowed for integrating electrodes in a concentric orbit around a waveguide within a single fibre^18,19^. Despite the advantage of the concentric arrangement in a thin fibre, this technique primarily has not been designed for constructing an array of the fibres. Moreover, a manual assembly was necessary for the arrangement of the fibres in a single platform to form a multi-shank array structure. In the case of a bifunctional optofluidic probe without the capability of electrical recording^21^, the size of a 500-μm-wide shank exhibited a limitation towards expanding to a multi-shank array form that would become too large to apply into a mouse brain. A multimodal microelectrode was fabricated through a micromachining process that included an epoxy-based manual bonding step^20^. But the incompatibility of the manual bonding with a wafer-scale process makes it considerably challenging to integrate into a multi-shank array structure. These limitations have prevented neuroscientists from investigating the functional connectivity between different regions in the intact brain. Moreover, the large size of the previously reported probes induces more significant tissue damage during the in vivo experiment.

**Fig. 1:**
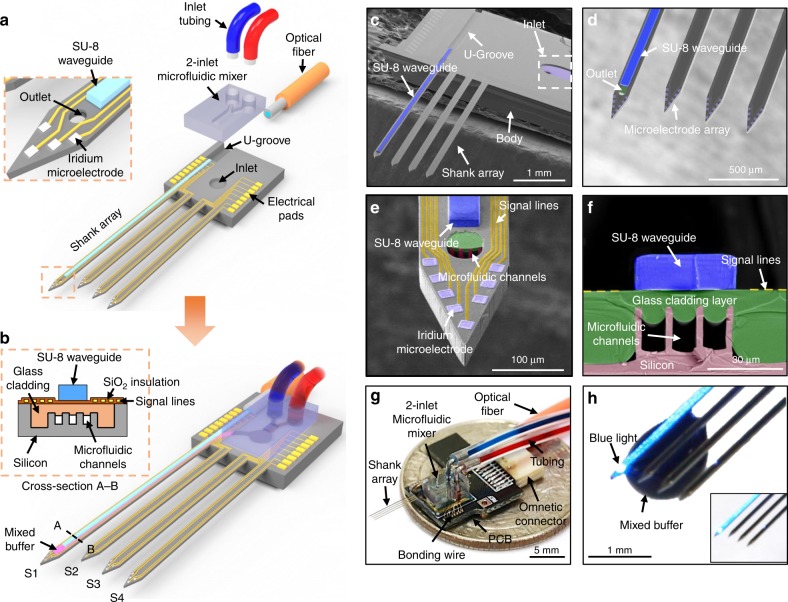
Fabricated multifunctional multi-shank MEMS neural probe: **a** Schematic diagram of the respective components before the device packaging with inset showing the probe tip; **b** Schematic diagram of the packaged device with inset showing the cross-section of the probe shank; **c** View of the multifunctional MEMS neural probe; **d** A magnified image of the shank array showing the integrated microelectrode array, the SU-8 waveguide, and the outlet; **e** Close-in image of a leftmost shank tip with the optical stimulation site and the outlet of the microfluidic channel; **f** Cross-sectional image of the leftmost shank showing the embedded glass cladding and microfluidic channels and the SU-8 waveguide; **g** Optical picture of the packaged multifunctional multi-shank MEMS neural probe; **h** A magnified image of the shank tip showing transmitted blue light through the SU-8 waveguide and the mixed liquid drug delivered through microfluidic channels

Herein, we present a multifunctional multi-shank MEMS neural probe that is monolithically integrated with (1) microfluidic channels for chemical delivery, (2) optical waveguides for optical stimulation and (3) microelectrode arrays for recording neural signals. This multi-shank structures enables ultimate mapping of long-range neural circuits between different regions of the brain at the cellular level (Fig. 1). This integration of the microfluidic channels and the optical waveguides is crucial because the capabilities of both optical stimulation and chemical delivery are essential in neural probe systems for studying the functional neural circuits in depth and for precisely modulating neural circuits in vivo. Our silicon micromachining-based fabrication offers an excellent advantage of scalability to a 2D microelectrode array, which enables the study of long-range neural circuits.

To integrate the two capabilities monolithically, we embed a glass layer that serves dual purposes. More specifically, it is a cladding layer for the waveguide and a supporting cover for the underlying microfluidic channels. Consequently, the dual-purposed glass layer allows for the construction of only 40-μm-thick probe shanks and then minimizes tissue damage during insertion owing to the six to eight times smaller cross-sectional area of our probe compared with other previously reported multifunctional probes^18–21^. Previously, we reported a microfluidic mixer, i.e. the staggered herringbone mixer (SHM), that was directly integrated on a neural probe and permitted the delivery of multiple drugs and changing their concentrations instantaneously with a small swept volume^22^. To demonstrate the potential applicability of our multifunctional multi-shank neural probe, we investigate the synaptic circuit between the hippocampal CA3 and CA1 regions in the intact brains of transgenic mice. The multi-shank multifunctional probe provides (1) simultaneous recording of neural signals in different regions, (2) modulation of long-range neural circuits through optical stimulation, and (3) drug infusion in the deep brain region of small animals. Our neurotechnology can provide various opportunities that facilitate studies of long-range neural circuits interconnected between regions of the brain, such as the hippocampal, thalamocortical, and amygdala circuits.

## Results

### Design of the multifunctional multi-shank MEMS neural probe

Our multifunctional multi-shank MEMS neural probe consists of four shanks (i.e., S1, S2, S3, and S4) (Fig. 1), and the lengths of all four shanks were greater than 7 mm, which allows the probe to reach any regions in the brains of the mice. The most critical consideration in designing the multifunctional probe was to integrate the two capabilities of optical and chemical modulations into a small form-factor. We integrated these two central features on a single shank (i.e. S1) for optical stimulation and chemical delivery. We also integrated an array of eight recording electrodes (20 × 20 µm⋅electrode^−1^) for monitoring the electrophysiological activities of neurons around the probe array. In this report, we primarily aimed to investigate the functional connectivity between the CA3 and CA1 regions in the hippocampus of mice by placing S1 and S2 into the CA3 region and placing S3 and S4 into the CA1 region. To provide complete coverage of the hippocampus, the space between each shank was 200 μm. The S1 was 200 μm longer than the other three shanks, so that it could be positioned at the left side of the CA3 region. Consequently, the S1 integrated with the dual features enabled optical and chemical stimulations in the CA3 region while monitoring neural signals both in the CA3 and CA1 regions.

For the chemical modulation, we designed a microfluidic staggered herringbone mixer (µSHM) with two inlets, which was integrated on the S1. This µSHM serves as an essential component in mixing two distinct fluids that exhibit laminar flow with low Reynold’s numbers^22^.

We integrated the µSHM to deliver into the target region of the brain either two different chemicals simultaneously or a chemical with real-time variations of concentration. We used polydimethylsiloxane (PDMS) that allowed for a compact and secure interface between the µSHM and the silicon body of our neural probe.

Our multifunctional neural probe, including microfluidic channels, a SU-8 optical waveguide, and eight electrodes on each shank, can be fabricated with the minimum width, thickness, inter-shank spacing of 128, 40 and 30 μm (shank-to-shank pitch of 130 μm), respectively. So, these small critical dimensions can lead to a variety of probe designs for various applications. Moreover, the length of each shank, as well as the position of electrodes, can be custom-designed depending on target locations (Supplementary Fig. 2). Therefore, our proposing shank-array structure provides wide design flexibility to accommodate target locations in the brain, sites for optical stimulation, chemical delivery and electrical recording.

### Fabrication and packaging of the multifunctional probe

We fabricated the multifunctional multi-shank neural probe based on MEMS technology that enabled small, thin mechanical structures with precise dimensions, including S1 with the dual features of optical and chemical modulations (Supplementary Fig. 1). Most notably, we embedded a glass layer in the silicon substrate for dual purposes by using the glass reflow process that we had developed previously^17,22,23^. The embedded glass layer exhibits high yield strength of 8.4 GPa, which is comparable to that of silicon (7 GPa)^24^. Thus, our probe consisting of both glass and silicon can endure high stress just as silicon-based neural probes do. Also, the embedded glass layer served as a thick cladding (la5 µm) layer for an optical waveguide^14^ and as a transparent cover for the parallel microfluidic channels on S1 (Fig. 1b), which led to the production of a thin probe shank (Fig. 1c–h). The width and the thickness of each shank were 128 and 40 μm, respectively. The mechanical stiffness of our multifunctional probe is comparable to previously reported flexible multifunctional probes^18–20^ because of its small form-factor despite much higher Young's modulus of silicon (Supplementary Table 1). The small form-factor enables reduced tissue damage during the insertion process, and we also expect that low bending stiffness help to reduce tissue damage in performing long-term in vivo experiments.

We used SU-8 (a negative photoresist) as a core layer of the optical waveguide^14^, and it was supported by the glass cladding layer (Fig. 1f). Because the refractive index of SU-8 (1.54) is higher than that of glass (1.46), light from an optical fibre can reach a stimulation site through the waveguide. To align the optical fibre (50-μm-wide core and 125-μm-wide cladding) to the waveguide, we patterned a U-groove on the body of the probe array (Fig. 1a, c). A cross-sectional view of S1 (Fig. 1f) confirmed that the glass layer supports the gold signal lines as well as the 40-μm-wide and 15-μm-thick SU-8 core layer of the optical waveguide and covers three 10-μm-wide and 12-μm-high microchannels. We fabricated the three parallel microchannels so that they merged into a shared 30-μm-wide, 12-μm-high outlet (Fig. 1e, f) instead of a single, wide channel to prevent the reflowed glass from overfilling and blocking the channel space.

We packaged the fabricated neural probe array for the in vivo experiments (Fig. 1g). We bonded the PDMS-based µSHM with two inlets using an O_2_-plasma treatment. An outlet of the µSHM was aligned with an inlet of the probe array so that the two different fluids that were mixed homogeneously in the µSHM were delivered through the microchannels in the S1. Then, we placed the optical fibre on the U-groove and fixed it there with UV-curable epoxy (NOA 148). Figure 1h shows an example operation of transmitting blue light as well as delivering blue-dyed and red-dyed buffer.

Because our fabrication and packing of neural probes are modular, the expansion to an array structure can be accomplished quite readily. The accurate control of the dimensions of the probe and the distance between shanks is vitally important in revealing the connectivities in and between various brain regions.

### Characterizations of the multifunctional probe

**Fig. 2:**
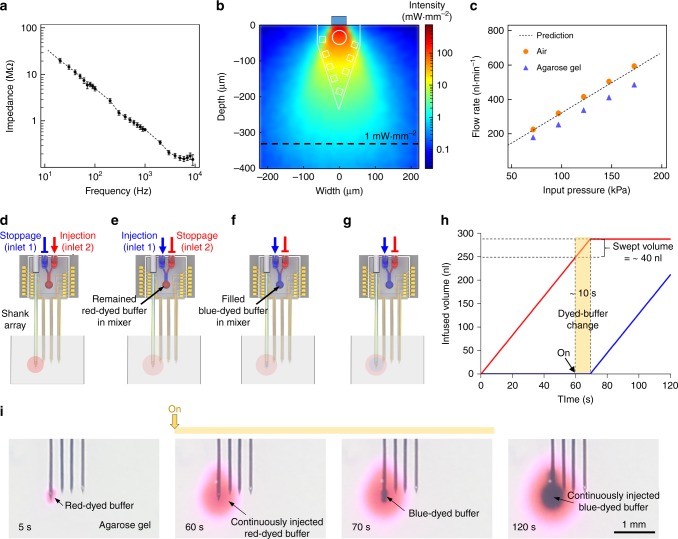
Characterization of the multifunctional multi-shank MEMS neural probe: **a** Impedance plot of 32 the Iridium microelectrodes. Error bars indicate s.d.; **b** Simulated light intensity map in brain tissue using Monte Carlo simulation based on measured output power (blue light, 454 μW at the end of the SU-8 waveguide); **c** Plot of flow rates with different input pressures; **d**–**g** Schematic diagram of multi-dyed buffer injection: **d** Loaded red-dyed buffer injection in inlet 2; **e** A stoppage of red-dyed buffer injection to the agarose gel and loaded blue-dyed buffer injection in inlet 1; **f** Blue-dyed buffer injection into the agarose gel after flushing out the filled red-dyed buffer dye in the microfluidic mixer; **g** Continuous infusion of the blue-dyed buffer into the agarose gel; **h** Plot of the infused volume of red- and blue-dyed buffers from the probe to 0.9% agarose gel; **i** Sequential photographs of multi-dyed buffer injection in 0.9% agarose gel when the flow rate was 0.3 μl⋅mim^−1^

To evaluate the performance of our multifunctional multi-shank MEMS neural probe before using it in in vivo experiments, we characterized three critical aspects associated with functions of our probe array, i.e., (1) the electrical impedance of the electrodes, (2) the optical power density near the waveguide and (3) the delivery of multiple chemicals. First, we measured the electrical impedance of the 32 iridium (Ir) microelectrodes (eight electrodes on each shank). The average impedance was 0.665 ± 0.043 MΩ at 1 kHz, which was sufficiently low to record neural activities^25^ (Fig. 2a).

The output power of light at a stimulation site should be higher than the threshold intensity of 1 mW⋅mm^−2^ to activate channelrhodopsin-2 (ChR-2)^26^. Thus, we measured the optical power of blue light (473 nm) transmitted from the end of the SU-8 waveguide, and we calculated the propagation and insertion losses between the optical fibre and the SU-8 waveguide. When the input optical power was 32 mW, the output optical power at the end of the SU-8 waveguide was 454 μW, which corresponded to an optical power density of 757 mW⋅mm^−2^. Besides, we calculated the light intensity using a numerical metohd^14,27–29^ and simulated the distribution of light using a Monte Carlo simulation (Monte Carlo eXtreme; MCX)^30,31^ to estimate light intensity from the SU-8 waveguide over all the eight electrodes on the shank that was implanted in the brain for the optical stimulation (More details are provided in the Method and Supplementary Note 1.). Based on both the measured optical power at the end of the SU-8 waveguide and the dimensions of the probe tip, the blue light propagated up to ~330 μm from the waveguide with a light intensity greater than the threshold intensity of 1 mW⋅mm^−2^ (Fig. 2b). We confirmed that sufficient light intensity ( c4 mW⋅mm^−2^) illuminated all of the recording electrodes near the SU-8 waveguide (Fig. 2b and Supplementary Fig. 3).

We evaluated the performance of the embedded microfluidic channels connected with the µSHM with which we have previously demonstrated the capability of multi-drug delivery^22^. First, we measured the flow rates through the microfluidic channels in the S1, either exposed to ambient air or inserted in an agarose gel, by applying input pressure (70–170 kPa) from the two inlets. We chose 0.9% [w/v] agarose gel, which represents a slightly denser matrix than the brain, to ensure the infusion and dispersion of the drug in the brain^32^. The flow rate was 350 nl⋅min^−1^ (in air) and 230 nl⋅min^−1^ (in agarose gel) at 100 kPa, which were similar to the flow rates calculated by hydraulic resistance of the microfluidic channels with various input pressures^17^ (Fig. 2c, Supplementary Fig. 4 and Supplementary Note 2). We also estimated the deliverable region by infusing 1 μl of a blue-dyed buffer into the agarose gel at 250 nl⋅min^−1^ for 4 for Time-lapse images indicated widespread dispersion over 1 mm covering all four of the shanks (Supplementary Fig. 5). The elliptical dispersion profile was due to the continuous convection in the out-of-plane direction.

For the sequential delivery of two different chemicals (e.g., red- and blue-dyed buffers), we estimated the swept volume, which is defined as the volume of the second chemical that was observed coming out of the inlet of S1 after initiating infusion from an inlet. After continuous infusion of the red-dyed buffer at a flow rate of 300 nl⋅min^−1^ for 60 s, the infusion was switched to the blue-dyed buffer. To estimate the residence time of the red-dyed buffer during a solute exchange to the blue-dyed buffer, we analysed the temporal evolution of the intensity of both red and blue channels at the in- and outlets (Fig. 2d–g). The intensity of the blue channel turned over that of the red channel in ~4 s at the inlet, and an almost complete turn-over occurred within additional 5 s (Supplementary Fig. 6; top panel). The turn-over appeared at the outlet in ~10 s, and the intensity of the blue channel continuously increase over 95 s (Supplementary Fig. 6; middle panel). These measurements indicate that the residence (flush) time between a reagent exchange was ~10 s (Fig. 2h, i and Supplementary Fig. 6; bottom panel). At the outlet, both blue and red intensity values reached a plateau much more slowly due to both dyes diffusing in the agarose gel near the outlet. The measured residence time of 10 s and the flow rate of 300 nl⋅min^−1^ corresponded to a total internal volume of ~40 nl in the µSHM and the microchannels in S1. These data indicated that we can change chemicals within ~10 s with the quite small swept volume.

### Immune responses by the multifunctional probe in the brain

We evaluated the mechanical invasiveness by assessing immune responses by our multifunctional probe. We inserted the probe and a stainless-steel wire with a cross-sectional diameter of 127 µm in the left and right hippocampus regions (coordinates: AP -1.34, ML ± 1.50, DV -1.80; millimetre relative to bregma) of the wild-type mouse (*n* o6, C57BL6; 10 weeks), respectively. The stainless-steel wire has been widely used for both recording neural signals and electrical stimulation. The cross-sectional area of the steel wire is more than two times smaller than that of previously reported multifunctional probes^18–21^. We compared the immune responses in the brain around our probe and the stainless-steel wire implanted for 6 h and 2 weeks.

We confirmed that both GFAP (reactive astrocyte)- and Iba1 (microglia)-positive signals, as cellular indicators for the immune response, around our MEMS probe in the brain remained almost identical over 2 weeks compared with 6 hr after implantation (Supplementary Fig. 7 and Supplementary Fig. 9). In contrast, the GFAP- and Iba1-positive signals became more prominent in 2 weeks with the stainless-steel wire implanted. These data suggest that similar or fewer astrocyte, macrophages, and microglia migrated around the MEMS multifunctional neural probe, compared with the stainless-steel wire through both short- and long-term implantation, which indicates that our multifunctional MEMS neural probe causes little immune responses over 2 weeks.

We reason that these minimized immune responses were attributed primarily to small dimensions of our probe leading to reduced insertion tissue damage (Supplementary Fig. 8). The cross-sectional area of our probe was 5120 μm^2^ whereas that of the stainless-steel wire was 12,668 μm^2^. We confirmed that the cross-sectional dimension of the implanting structure determined the size of the damaged region. We also found that the cross-sectional area of our probe was significantly smaller by a factor of 6–8 than that of other multifunctional neural probes (Supplementary Table. 1).

Furthermore, we examined the immune responses by the optical stimulation for a short term with the probe implanted in the same hippocampus region of a wild-type mouse (*n* u 3, C57BL6; 10 weeks). We applied the optical stimulation (1 Hz with a 50% duty cycle, 167 mW⋅mm^−2^ with a total power of 100 μW) for the first 1.5 h after implantation. Based on the immunostaining of both astrocytes (GFAP) and microglia (Iba1) at 6 h after implantation (i.e. 4.5 hr after the optical stimulation), little differences in the GFAP- and Iba1-positive signals appeared (Supplementary Fig. 10). These data suggest that the optical stimulation by the stimulation condition with our multifunctional MEMS neural probe affects little additional immune responses that can ultimately lead to phototoxicity.

### Localized optical stimulation with the multifunctional probe

For investigating the functional connectivity between regions in the brain, it is essential to stimulate one or more regions locally by electrical, chemical, or optical stimulation and to monitor neural activities in adjacent regions. Therefore, we first confirmed the capability of providing localized optical stimulation by inserting the probe array into both the hippocampal CA1 and the somatosensory cortex (Supplementary Fig. 11a). We chose those two regions intentionally because they are known to be connected unidirectionally from the somatosensory cortex to the hippocampus^33–35^ rather than the other way around.

When the S1 was placed in the CA1 (coordinates: AP -1.70, ML -1.50, DV -0.75; millimetre relative to bregma) and the other three shanks (i.e. S2, S3 and S4) in the somatosensory cortex (Supplementary Fig. 11a) of the anesthetized transgenic mice (*Thy1-ChR2-eYFP*, *n* 2) (More details are provided in the Methods section.) Spontaneous signals appeared simultaneously from all four of the shanks with noise levels of ~±15 μV. After stabilization for 30 min, we stimulated the CA1 through the optical waveguide on the S1 while recording the neural signals from the four shanks. We observed neural activities that were evoked by as well as synchronized by light pulses (1 Hz with a 50% duty cycle, 167 mW⋅mm^−2^ with a total power of 100 μW) only from the S1 (Supplementary Fig. 11b). However, no signals were observed that were correlated with the light pulses (Supplementary Fig. 11b). Note that we show this uncorrelated activity of somatosensory cortical neurons from the S2 as the closest shank that could have been affected by the light pulses from the S1.

For the precise analysis of the neural signals, we sorted spikes with a customized MATLAB algorithm (Supplementary Code 1). Raster plots of the sorted neural signals, acquired from 25 optical stimulation trials, also indicated quantitative differences in the firing rate of the hippocampal neurons (Supplementary Fig. 11c). However, the firing rate of the somatosensory cortical neurons remained unchanged (Supplementary Fig. 11d). These data confirmed that light delivered through the waveguide on the S1 only stimulated neurons that were near the stimulation site (i.e. ea328 μm); the shank-to-shank distance was 328 μm. Therefore, our multi-shank MEMS neural probe enabled localized optical stimulation.

### Investigation of hippocampal CA3-CA1 connectivity in vivo

**Fig. 3:**
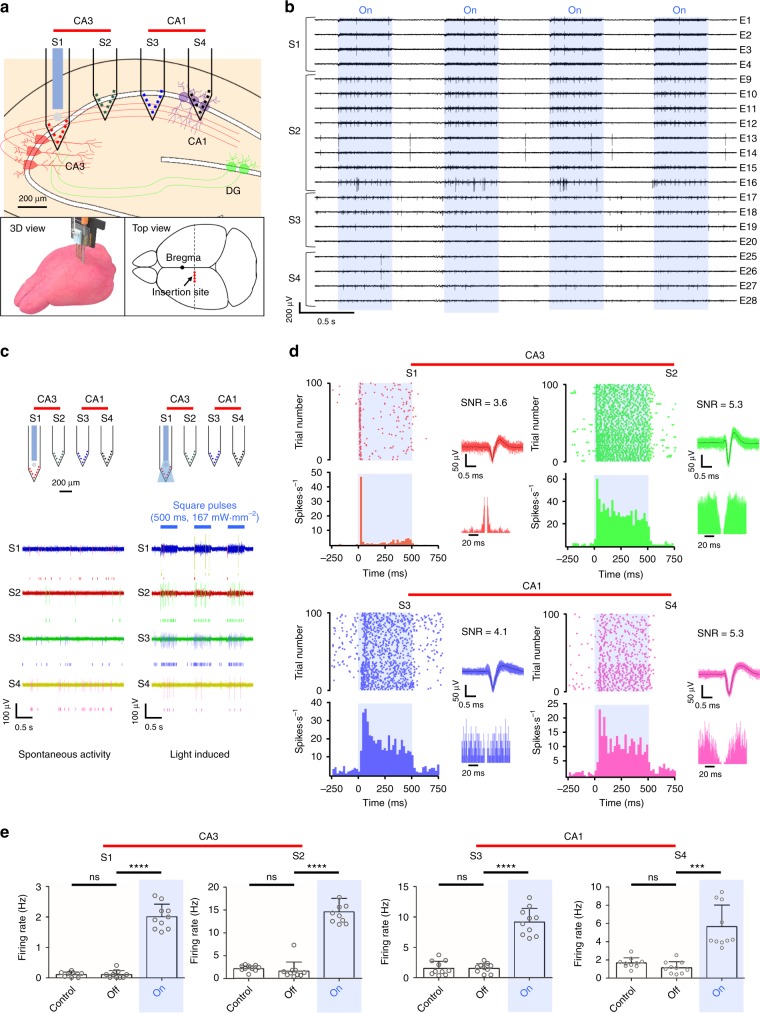
Investigation of the connectivity through neural recording and optical stimulation: **a** The multifunctional multi-shank MEMS neural probe inserted in CA3 and CA1 of the hippocampus of transgenic Thy1-ChR2-YFP mice; **b** Neural signals recorded from the electrodes in shank 1, 2 inserted in hippocampal CA3 and shank 3, 4 inserted in hippocampal CA1 during light stimulation (1 Hz, 50% duty cycles; blue background indicates the onset of light); **c** Representative raster plot of sorted neural signals from spontaneous activities before optical stimulation and light-induced activities during optical stimulation (blue mark indicates light onset); **d** Representative raster plots of detected neural signals from electrodes in hippocampal CA3 and CA1 during off and on cycles and peristumulus time histograms (PSTH) of 100 trials and sorted neural spike signals with the autocorrelograms and SNR of the sorted signals; **e** Comparison of the firing rate of the spontaneous activity (control), off cycle during optical stimulation and on cycle during optical stimulation show the connectivity between hippocampal CA3 and CA1. Error bars indicate s.d. (S1: ns *P* s 0.9999, *t* 90(Control-Off), ****P **0.0001, *t* 013.83(Off-On), S2: ns *P* ns0.4475, *t* t40.7766(Control-Off), ****P **0.0001, *t* t011.49(Off-On), S3: ns *P* ns0.9823, *t* t90.02252(Control-Off), ****P *0.0001, *t* t010.15(Off-On), S4: ns *P* ns0.0737, *t* t01.899(Control-Off), ***P **0.0001, *t* t05.755(Off-On). d.f. .f18 and *n* 8 10 for all, *n* firing rate for 10 trials, Two-tailed *t*-test)

We investigated the functional connectivity in the intact brain of mice with our multifunctional multi-shank MEMS neural probe. We chose the CA3-CA1 as one of the well-known trisynaptic circuits in the hippocampus because there have been extensive studies of the functional connections between these two regions via Schaffer Collaterals^36–38^. We implanted the neural probe array in the hippocampus (coordinates: AP -1.34, ML -1.50, DV -1.80 at the location of the S1; millimetre, relative to bregma) of the transgenic mice (*Thy1-ChR2-eYFP*, *n* 4); the S1 and S2 were placed in the CA3, and the S3 and S4 were placed in the CA1 (Fig. 3a and Supplementary Fig. 16). After ascertaining spontaneous neural activities in both regions, we used blue light (473 nm) via the optical waveguide on the S1 to stimulate CA3 neurons (1 Hz with a 50% duty cycle, 167 mW⋅mm^−2^ with a total power of 100 μW) while recording neural signals from the 32 microelectrodes on the four shanks (Fig. 3b). Then, we applied a bandpass filter (0.3–6 kHz) to the recorded neural signals that included spikes that were sorted based on their amplitudes and shapes using our custom MATLAB code (Supplementary Code 1).

Notably, we were able to observe CA3 spikes that were evoked directly by square pulses of the blue light and CA1 spikes synchronized with the CA3 signals (Fig. 3c, d, Supplementary Fig. 12). More detailed analyses with raster plots of the sorted neural signals from 100 trials, as well as their corresponding peri-stimulus time histograms (PSTHs), reaffirmed that the CA1 pyramidal neurons were excited almost simultaneously by the synaptic transmissions from CA3 to CA1 (Fig. 3d and Supplementary Fig. 13). Accordingly, the firing rate of both the CA3 and CA1 neurons increased dramatically when there was local optical stimulation of the CA3 region (Fig. 3e).

### Modulation of hippocampal CA3-CA1 connectivity in vivo

**Fig. 4:**
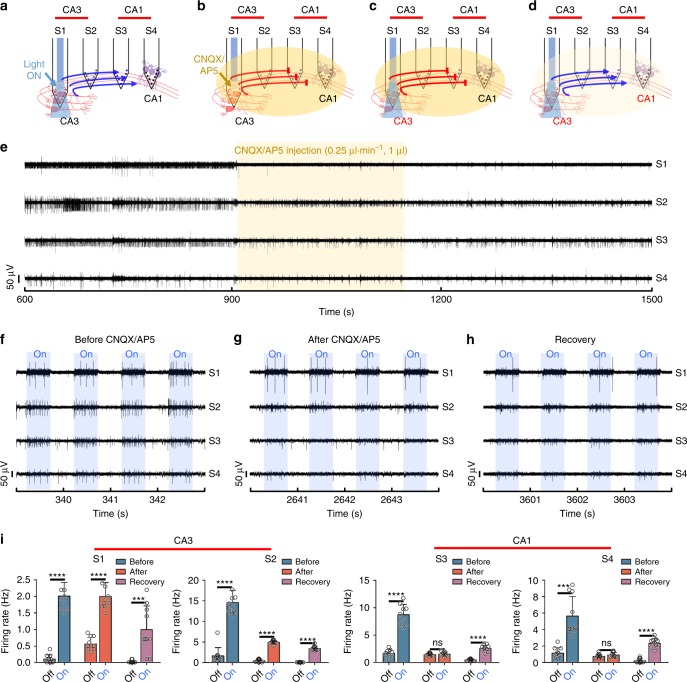
Modulation of the neural circuit through the synapse blockage with drug delivery: **a**–**d** Conceptual diagrams of the experimental process: **a** The connectivity verification between hippocampal CA3 and CA1 by optical stimulation; **b** The synapse blockage between hippocampal CA3 and CA 1 by CNQX and AP5 injection; **c** The verification of blocking connectivity between hippocampal CA3 and CA1 by optical stimulation; **d** Recovery of connectivity by drug diffusion in the brain; **e** Representative transient plot during CNQX and AP5 injection at shank 1 (yellow mark indicates drug injection); **f**–**h** Transient plot during optical stimulation before and after CNQX and AP5 injection and recovery (the blue mark indicates light onset); **i** Comparison of the firing rate of the off cycle during optical stimulation and the on cycle during optical stimulation before and after CNQX and AP5 injection and recovery in hippocampal CA3 and CA1. Error bars indicate s.d. (S1: ****P < 0.0001, *t* t013.83(Before), ****P **0.0001, *t* t09.737(After), ***P *0.0004, *t* t04.337(Recovery), S2: ****P  **0.0001, *t* t011.49(Before), ****P *0.0001, *t* t024.83(After), **** *P* *0.0001, *t* t019.7(Recovery), S3: ****P **0.0001, *t* t010.15(Before), ns *P* n0.5657 *t* t50.5852(After), ****P *0.0001, *t* t010.71(Recovery), S4: ***P *0.0001, *t* t05.755(Before), ns *P* n0.2046, *t* t21.316(After), ****P *0.0001, *t* t010.69(Recovery). d.f. .f18 and *n* 8 10 for all, *n* 0 firing rate for 10 trials, Two-tailed *t*-test)

To demonstrate the modulation of the functional connectivity via the synaptic transmission between CA3 and CA1, we injected α-amino-3-hydroxy-5-methyl-4-isoxazolepropionic acid (AMPA) receptor antagonist cyanquixaline (CNQX); 6-cyano-7-nitroquinoxaline-2,3-dione; 20 μM, 0.25 μl⋅min^−1^, 1 μl and N-methyl-D-aspartate (NMDA) receptor antagonist AP5 ((2 *R*)-amino-5-phosphonovaleric acid; 50 μM, 0.25 μl⋅min^−1^, 1 μl) through the microfluidic channels that were embedded in our probe array (Fig. 4a). We expected that the receptor antagonists that were delivered to CA1 would block the synaptic transmission and that the neural activities in CA1 evoked by the optical stimulation in CA3 would no longer be observed (Fig. 4b). When we illuminated the blue light, neural activities of both the CA3 and CA1 neurons synchronized with the light pulses as identically as occurred in the previous experiments demonstrated above (Fig. 4f).

After the injection of CNQX and AP5 to CA3 for 4 min, we optically stimulated neurons in the CA3 region 20 min later for the sufficient spread and reaction time of two receptor antagonists from CA3 to CA1 (Fig. 4b, e). When CA3 was stimulated optically (Fig. 4c), the synchronized neural activities of the CA1 neurons decreased significantly despite the persistently increased activities of the CA3 neurons (Fig. 4g); this was due to the fact that the excitatory pathway between CA3 and CA1 was blocked by the antagonists delivered through the S1. We also compared neural activities in both CA1 and CA3 before and after the optical stimulation and after the injection of the antagonists. Firing rates estimated from stacked raster plots and PSTHs were in excellent agreement with the temporal evolutions of the spikes recorded from our probe array (Fig. 4i and Supplementary Fig. 14-15).

Interestingly, the CA3-CA1 circuit was recovered, to a substantial extent, in 35 min after the injection of CNQX and AP5, although the amplitude and firing rate of the recorded neural signal were not fully recovered due to residual CNQX and AP5 (Fig. 4d, h, i). The synaptic transmission of the CA1 neurons, which was blocked previously, started showing responses synchronized with the optical stimulation to CA3 (Fig. 4h, i). This reoccurrence of the synchronized activity was attributed to the concentration of the antagonists, which was diluted substantially by diffusion away from the S1 through the brain. The extent of the firing rate decreased markedly after the recovery during the optical stimulation (i.e. on-state) in both CA3 and CA1. The neural activity in CA1 was due to the decreased firing of the CA3 neurons. These data indicate that the connectivity in CA3 could consist of multiple neurons, and, thus, the intermediate synaptic transmissions still were blocked by the residual, non-negligible antagonists.

To summarize, by delivering CNQX and AP5 through the microfluidic channels, we were able to use our multifunctional multi-shank neural probe to successfully modulate the hippocampal excitatory pathway between the CA3 and CA1 pyramidal neurons.

## Discussion

Herein, we presented a multifunctional multi-shank MEMS neural probe that is monolithically integrated with (1) an optical waveguide for optical stimulation, (2) microfluidic channels for chemical delivery and (3) microelectrode arrays (32 electrodes) for recording neural signals and mapping neural circuits at the cellular level. An array structure with accurately defined spacing between probe shanks provided a crucial advantage in studying neural circuits. This array structure was particularly advantageous for studying long-range neural circuits. Also, the various stimulation capabilities with the array structure enabled the confirmation of the functional connectivities and the modulation of the neural circuits. In addition, the small (micrometre-scale) probe structure can significantly reduce tissue damage when the probe is inserted into the brain. The minimal tissue damage, in the presence of the probe implanted in the brain, also was an essential aspect for chronic recordings and modulations.

We demonstrated that the multifunctional multi-shank MEMS neural probe could be used to confirm the connectivity between the hippocampal CA3 and CA1 regions of the mouse brain by the optical stimulation in CA3 while recording spikes in both CA3 and CA1. To modulate the neural circuit between CA3 and CA1, we delivered receptor antagonists, which blocked the post-synaptic transmission. The array structure presented in this report can be scaled readily and can be used in more complex circuit studies where three or more brain regions are connected, which has not been possible to date using prior systems^18,21^.

The packaging of neural probe systems also has been a substantial barrier to the use of the systems in various neuroscience applications. We developed a simple microfluidic interface with PDMS in order to deliver various chemicals. The self-alignment between an optical fibre and the waveguide helped simplify the packaging process without any complicated optical equipment. However, the large coupling loss between the optical fibre and the waveguide can be reduced by either integrating an interfacial optical component such as GRIN lens^39^ or by employing an optical fibre with a smaller size and high NA (e.g. 44-μm-radius-core, 0.66 NA provided by Doric lens). The total weight of the packaged system is less than 1.5 g, including the optical and fluidic interfaces and an electrical connector. The size and the weight of our system are suitable for uses in small animals, such as mice, but the system should be advanced with a wireless interface to extend its applicability in other areas, such as social behavior studies^40–42^. The integration of a small pumping system and light sources would further reduce the size and enhance the versatility of our multifunctional neural probe system.

## Methods

### Fabrication of a multifunctional MEMS neural probe

To fabricate the multifunctional MEMS neural probe, we used a 4-inch silicon-on-insulator (SOI) wafer with a 40-μm-thick top silicon and 0.7-μm-thick buried-oxide layer. First, two side cavities (25-μm high and 25-μm wide) and three centre cavities (25-μm high and 10-μm wide) were formed by etching the top silicon substrate using deep reactive ion etching (DRIE) with both photoresist as the soft mask and silicon oxide as the hard mask (Supplementary Fig. 1). After removing the soft mask, the top silicon substrate was etched additionally by 10 μm. Then, the hard mask was removed by buffered oxide etchant (BOE). After cleaning the etched silicon wafer by piranha cleaning, a 500-μm-thick borosilicate glass wafer (Borofloat^®^33) was bonded anodically under vacuum to a silicon wafer with etched cavities. The bonded glass was thinned by 100 μm using chemical mechanical polishing (CMP) followed by reflow of the glass at 750 °C for 90 min at atmospheric pressure using rapid thermal annealing (RTA)^43,44^. The glass in the side cavities reflowed faster than the glass in the centre cavities because of the difference in their volumes. Thus, by controlling the temperature and the process time, we were able to control the height of the microfluidic channels precisely with higher process margin. Hence, the glass reflowing process produced an embedded glass layer that provided both embedded microfluidic channels and a thick cladding layer. After the unwanted glass was removed using chemical mechanical polishing (CMP), the planarized silicon wafer with the embedded glass layer was available for additional fabrication processes.

After depositing a 400-nm-thick silicon dioxide (SiO_2_) insulation layer using plasma-enhanced PECVD, 300-nm-thick gold and 20-nm-thick chrome adhesion layers were deposited as signal lines by sputtering and being patterned by ICP etching. Next, a 400-nm-thick SiO_2_ insulation layer was deposited to protect the signal lines and the contact areas for the microelectrodes were opened by etching the insulation layer using reactive ion etching (RIE). Microelectrodes (19 μm by 19 μm) were patterned, and a 20-nm-thick titanium (Ti) layer and a 150-nm-thick Ir layer were deposited followed by the lift-off process.

After pattering a 15-μm-thick SU-8 core layer on the glass cladding layer, a U-groove for placement of multimode optical fibre (125-μm-diameter) was patterned by successive etching processes using DRIE and RIE. The groove was etched by 55 μm for vertical alignment between the centre of the 125-μm-wide optical fibre and the centre of the 15-μm-high SU-8 waveguide. The front and back sides of the silicon wafer were patterned by photoresist for the shape of the shank and the formation of the inlet and outlet. The back and front sides of the SOI wafer were etched to the BOX layer using DRIE^45^. Then, the remaining BOX layer was etched from the back using RIE for complete isolation of the probe’s shank, followed by the removal of the remaining photoresist.

### Packaging of the multifunctional probe

The fabricated probe was packaged to provide a fluidic connection and a polydimethylsiloxane (PDMS) interface provided this connection between the inlet of the fabricated probe and the drug delivery system. Mixed PDMS with a 10:1 ratio of the base and curing agent was poured in the SU-8 master mold and cured at 80 °C for 1 h. The fabricated PDMS interface was bonded with the body of the probe by O_2_ plasma using a vacuum plasma system (Covance-MP, Femto Science, Korea).

The probe was glued on a custom printed circuit board (PCB) using a fast curing epoxy in order to provide an electrical connection. The electrical pads on the probe body were wire-bonded to the pads on the PCB, and an omnetic connector was soldered to provide electrical connections between the probe and the Neuralynx recording system (Digital Lynx 4SX System, Neuralynx, Bozeman, Montana, USA).

To provide an optical interface between the light source and the optical stimulation sites, we used a multimode optical fibre (GIF50, Thorlabs, Newton, NJ, USA) that was composed of a 50-μm-diameter core and 125-μm-diameter cladding layer and terminated FC/PC connectors. One end of the optical fibre was connected to a light source (ADR-2301, RGBlase LLC, Fremont, CA, USA), and the other end was placed on the U-groove of the probe to achieve coupling between the SU-8 waveguide and the fibre using the end-fire coupling. We manually aligned the fibre under a microscope using a mechanical xyz-stage. After aligning the fibre on the U-groove, we filled the gap between the optical fibre and the core of the SU-8 waveguide using UV-curable epoxy (NOA 148, Norland Products, Inc., Cranbury, NJ, USA) for both matching refractive index and fixing the fibre. Then, biocompatible black epoxy (EPO-TEK 320, Epoxy Technology, Inc., Billerica, MA, USA) was applied to block any leaking light from the junction between the optical fibre and SU-8 waveguide on the probe body.

### Electrochemical impedance

To perform electrochemical impedance spectroscopy (EIS), the microelectrodes of the probe (a working electrode) and a saturated calomel electrode as the reference electrode (CHI 151, CH Instruments, Inc, Austin, TX, USA) were immersed in 0.1 M phosphate-buffered saline (PBS) as the electrolyte. Then, we measured the impedance of the iridium microelectrodes with a frequency sweep (10 Hz-10 kHz) using an impedance analysis system (nanoZ, Neuralynx, Bozeman, Montana, USA)

### Optical properties

We coupled a photodetector (918D, Newport Inc., Irvine, CA, USA) to an optical power meter (1936-R, Newport Inc., Irvine, CA, USA) to characterize the power of the light output from the stimulated site. Then, the end of the probe was placed near the photodetector to measure the light intensity. The fluctuation in output power was within ±0.002 mW.

### Simulation on light distribution inside the brain

To estimate a light distribution from the SU-8 waveguide over all the eight electrodes on the shank for the optical stimulation within a complex environment such as the brain, we used the Monte Carlo simulation (Monte Carlo eXtreme; MCX)^30,31^. We simulated a brain tissue using a domain size of 100 × 100 × 100 with 0.02 mm voxels. We applied 0.02 (mm^−1^) of an absorption coefficient, 7.2 (mm^−1^) of scattering coefficient tissue, 0.89 of anisotropy and 1.36 of a reflective index to the brain tissue. A light source was located at voxel (50, 50, 1) with a light angle of 21.1º. A total of 1.12 × 10^12^ photons⋅ms^−1^ corresponding to 454 µW at 473 nm were emitted from the light source.

### Performance evaluation of microfluidic channels

To confirm the functionality of the microfluidic channels of the probe, we connected the probe to mass flow controllers (MFC, National Instruments), which connected to a nitrogen gas that provided air pressure. Also, we used a 23-gauge needle as an adaptor between the Tygon microbore tubing (ID: 0.05 cm, OD: 0.15 cm; S-54-HL) and the PDMS interface bonded to the body of the probe. We characterized the injection rate through our pressure-driven liquid delivery system in order to control the flow rate precisely. By injecting 0.1 M PBS through the microfluidic channels of the probe, we measured the movement distance of 0.1 M PBS in the tubing as a function of the pressure.

To measure the required time for exchanging liquid from inlet 1 to inlet 2, we first connected tubings filled with red- and blue-dyed buffers to the probe. After inserting the probe into a 0.9% [w/v] agarose gel, we injected the red-dyed buffer for 60 s through inlet 1 by the pressure-driven flow while pressure to inlet 2 remained zero. Subsequently, the buffer exchange from red to blue was initiated through inlet 2. Throughout the delivery of the red-dyed buffer, the buffer exchange, and the delivery of the blue-dyed buffer over 100 s, images were acquired, and then split into red (R), green (G), and blue (B) channels in ImageJ to calculate normalized mean intensity of R and B at both inlet 1 (point 1) and inlet 2 (point 2). By plotting temporal evolutions of the red and blue profiles at the two points, we estimated the required time for flushing out the microchannel as well as the swept volume (the residence time times the flow rate).

### In vivo electrophysiology

All of the procedures that involved the use of mice were approved by the Korea Institute of Science and Technology (KIST) in Seoul, Korea, and the procedures were conducted in accordance with the ethical standards stated in the Animal Care and Use Guidelines of KIST. The multifunctional multi-shank MEMS neural probe was tested in an adult male transgenic mouse (C57BL6 Thy1-ChR2-YFP; 10 weeks) and an adult male wild-type mouse (C57BL6; 10 weeks). The mice were anesthetized with 0.5% urethane (400 mg⋅kg^−1^, intraperitoneal injection). After positioning the anesthetized mice on a stereotaxic device (David Kopf Instruments, USA), the skin was removed and the skull was drilled near the target area based on the atlas of Paxinos and Franklin^46^. For histological verification of traces, the rear of the packaged probe was coated with DiI (V22885, Molecular Probe, USA), after which the probe was inserted into the hole made by the drill and lowered slowly down to the target region.

For optical stimulation in Thy1-ChR2-YFP mice, we used a laser source (ADR-2301, RGBlase LLC, Fremont, CA, USA). Stimulation frequencies were controlled using an optical chopper (square wave, 1 Hz, duty cycle of 50%). The drugs were delivered into the brain by injecting them through the microfluidic channels of the probe using our pressure-driven liquid delivery system.

Signals were recorded using Neuralynx (Digital Lynx 4SX System, Neuralynx, Bozeman, Montana, USA). The raw signals were filtered and digitized through the Neuralynx software (30 kS s^−1^ per channel, two sets of bandpass filter parameters: 0.3–6 kHz for LPF and 0.3–6 kHz for individual neural spike signals).

We used MATLAB software (The Mathworks, USA) to analyse the recorded signals. We used a custom spike-sorting algorithm to detect and sort spikes through signal polarity, negative and positive multiple amplitude thresholds for clustering the same signals, and a dead time of 1 ms for exclusion of double detection. The signal-to-noise ratio was calculated by dividing the mean of the peak amplitude of the detected signals by three times the standard deviation of the background noise^18^. Each spike was counted and expressed on a raster and bar plot for quantitative analysis of the change of the firing rate by the light. The significance of the results was assessed by the Student`s t-test using GraphPad Prism.

### Immune response test

To assess immune responses of the probe in the brain, we implanted both the multifunctional MEMS neural probe and a stainless-steel wire with a cross-sectional diameter of 127 μm (40944, Alfa Aesar, Haverhill, MA, USA) into the brain of an adult male wild-type mouse (C57BL6; 10 weeks). We observed activated astrocyte through glial fibrillary acidic protein (GFAP) and activated macrophages and microglia through ionized calcium-binding adaptor molecule 1 (Iba1) around the probe and stainless-steel wire. In vivo experiments to assess the immune responses were performed for 6 hr (short term) and 2 weeks (long-term). We note that the implanted probe was fixed with dental cement for the long-term experiment. Anesthetized mice were sacrificed at each time point by transcardial perfusion of 4% [w/v] paraformaldehyde (PFA) in 0.1 M PBS. After we carefully removed the probe, the extracted brain was fixed in 4% [w/v] PFA in 0.1 M PBS for 24 h at 4 °C. The fixed brain was cryoprotected with 30% [w/v] sucrose in 0.1 M PBS for 48 hr at 4 °C. Then, the cryoprotected brain was sectioned in the horizontal plane into 50 μm-thick slices. The 50 μm-thick brain slices were washed in 0.1 M PBS and blocked in a blocking solution containing 0.1% [v/v] Triton X-100 and 3% [w/v] bovine serum albumin (BSA) in 0.1 M PBS for 1 h at room temperature. After washing three times in 0.1 M PBS for 30 min, the 50 μm-thick brain slices were incubated with primary antibodies (mouse anti-GFAP, 1:500, ab10062, Abcam; goat anti-Iba1, 1:200, ab48004, Abcam) in 0.1 M PBS containing 3% [w/v] BSA for 24 hr at 4 °C. After washing three times in 0.1 M PBS for 30 min, the primary antibody-conjugated brain slices were incubated with secondary antibodies (goat anti-mouse conjugated Alexa Fluor 488, 1:500, ab150113, Abcam; donkey anti-goat conjugated Alexa Fluor 594, 1:200, ab150136, Abcam) in 0.1 M PBS containing 3% [w/v] BSA for 2 h at room temperature. After washing three times in 0.1 M PBS for 30 min, the brain slices were incubated with 4’,6-diamidino-2-phenylindole (DAPI; 1:1,000, D1306, Invitrogen) in 0.1 M PBS containing 3% [w/v] BSA for 1 h at room temperature. After finally washing three times in 0.1 M PBS for 30 min, the brain slices were mounted on glass slides. All the slices were imaged through a ×20 objective lens on a confocal microscope (LSM 700, Carl Zeiss, Oberkochen, Germany) with identical microscope settings, and, we analysed fluorescent images using line profile and oval profile functions of imageJ.

### Histology

Immediately after the electrophysiological experiments, the mice were perfused transcardially with physiological saline followed by 4% paraformaldehyde in 0.1 M PBS. After overnight postfixation in the same fixative, the brains of the mice were immersed with a 30% sucrose in 0.1 M PBS for 24 h. Subsequently, the brains were cut into 300-μm coronal sections using a sliding microtome. The sections were mounted on cover slides with a mounting medium. Sections were observed in a fluorescent microscope to confirm probe tracks.

### Statistical analysis

All analyses were performed using MATLAB (Math Works) and GraphPad Prism (GraphPad Software). The statistical analyses were performed using the Student`s *t*-test.

### Reporting summary

Further information on research design is available in the Nature Research Reporting Summary linked to this article.

## Supplementary information

Supplementary Information

Description of Additional Supplementary Files

Supplementary Code 1

Reporting Summary

## Data Availability

The data that support the findings of this study are available from the corresponding author upon reasonable request.

## Code availability

The code used for the spike-sorting process is freely available on Supplementary Code 1. This code was used with MATLAB 2016a version.
