# Supplementary material for: Multifunctional multi-shank neural probe for investigating and modulating long-range neural circuits in vivo
Source: Nat Commun. 2019 Aug 22;10:3777. doi: 10.1038/s41467-019-11628-5 (PMC6706395; doi:10.1038/s41467-019-11628-5)
Supplement: Supplementary file 1 — Supplementary Information [file 41467_2019_11628_MOESM1_ESM.pdf]

Supplementary Information:

Multifunctional multi-shank neural probe for investigating and modulating long-range neural circuits *in vivo*

Shin et al.

	**Multifunctional MEMS probe (This work)**	**Multifunctional fiber (Park et al., 2017)**	**Optofluidic probe* (Jeong et al., 2015)**	**Multimodal microelectrode (Birthe et al., 2013)**
Array structure	Yes	No	No	No
Function	Electrical recording<br>Optical stimulation<br>Fluidic delivery	Electrical recording<br>Optical stimulation<br>Fluidic delivery	-<br>Optical stimulation<br>Fluidic delivery	Electrical recording<br>Optical stimulation<br>Fluidic delivery
Dimensions**		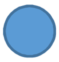	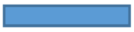	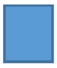
	128 μm in width<br>40 μm in thickness	200 μm in diameter	500 μm in width<br>80 μm in thickness	190 μm in width<br>225 μm in thickness
Area	5,120 μm^2	31,400 μm^2	40,000 μm^2	42,750 μm^2
Mechanical property of substrate	Silicon	Polycarbonate	PDMS	SU-8
	130 GPa	2.38 Gpa	1 Mpa	2 GPa
Stiffness	8.87×10^{-8} Nm^2	1.87×10^{-7} Nm^2	8.33×10^{-10} Nm^2	3.61×10^{-7} Nm^2

* Optofluidic probes were implanted into the brain using 50 μm stainless steel wire as an insertion shuttle.
** The cross-sectional diagram of the probes is drawn to scale.

Supplementary Table 1. Comparison of specifications of previously reported neural probes. The bending stiffness K is calculated using dimensions and mechanical property listed in Supplementary References[1-3]. For rectangular probes, $K = E \times \pi w t^3/12$, where *E* is the Young`s modulus of the substrate material of a probe, *t* and *w* are the thickness and the width of the probe, respectively. For cylindrical probes, $K = E \times \pi d^4/64$, where *d* is the diameter of a probe.

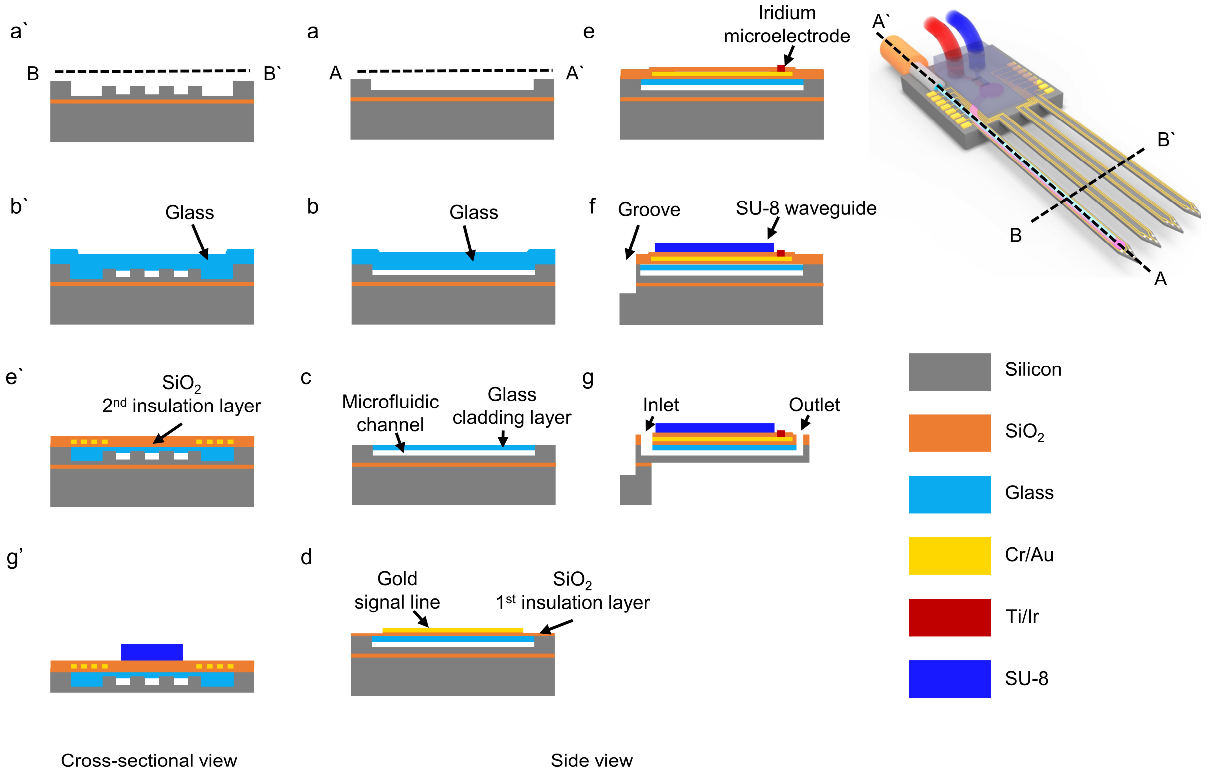

Supplementary Figure 1. Fabrication process flow of a multifunctional probe. (a) Formation of cavities for a microfluidic channel; (b) Anodic bonding between a glass wafer and patterned SOI wafer followed by the thermal reflow process using RTA; (c) Removal of the unwanted glass layer using CMP; (d) Deposition of SiO_2 insulation layer for passivation between silicon and Cr/Au signal lines; (e) Deposition of SiO_2 insulation layer for protecting signal lines; (f) Patterning of SU-8 core layer on the glass cladding layer followed by etching of silicon for forming U-groove; (g) Isolation of shank by etching silicon from the back using DRIE process.

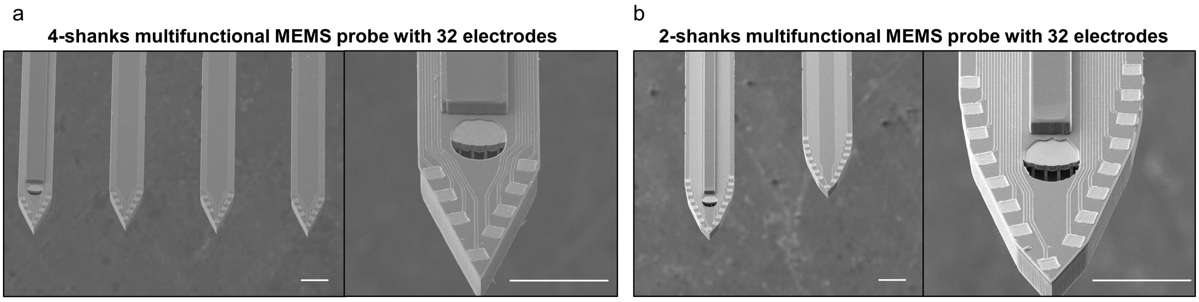

Supplementary Figure 2. SEM images of two different probe designs: (a) Multifunctional MEMS probe with four shanks of the same length and 32 electrodes and (b) Multifunctional MEMS probe with two shanks of the different lengths separated by 250 µm (scale bars = 100 µm).

a b

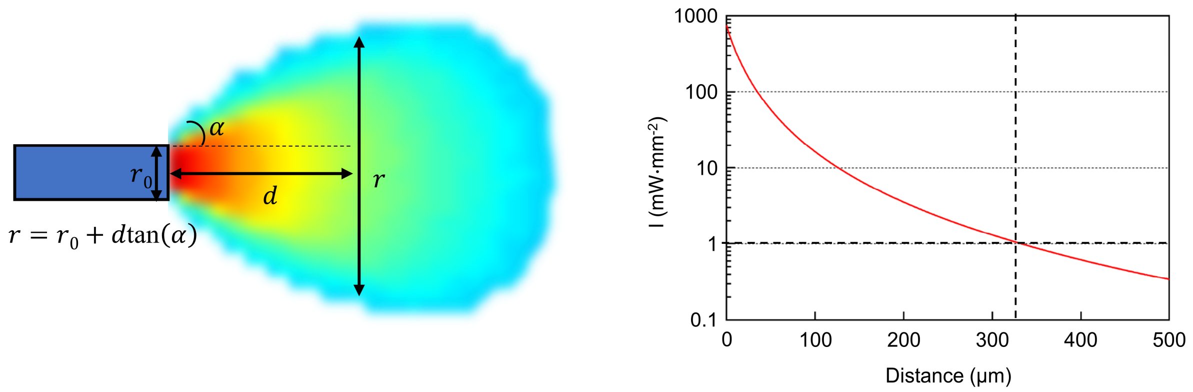

c

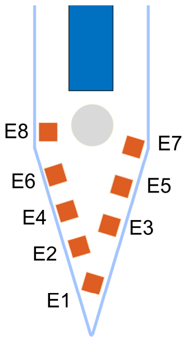

Electrode number	Distance (μm)	Estimated intensity (mW·mm^{-2})	Simulated intensity (mW·mm^{-2})
E8	60	42.99	5.75
E7	70	32.68	27.63
E6	83	23.74	23.24
E5	95	18.21	20.89
E4	115	12.29	13.66
E3	125	10.29	11.34
E2	150	6.89	7.25
E1	185	4.26	4.56

Supplementary Figure 3. Estimation of light intensity in brain. (a) Scheme of the light propagated along the distance from the waveguide in the brain tissue. Our waveguide was a rectangular structure (width of 40 μm and thickness of 15 μm), so an equivalent radius was computed (i.e. r_0=13.82 μm); (b) Calculated light intensity along the axial distance from the end of the SU-8 waveguide (blue light, 100 μW at the end of SU-8 waveguide) using equations for power-intensity transformation. (c) Calculated and simulated light intensity by equations for power-intensity transformation and Monte Carlo simulation[4] on each electrode.

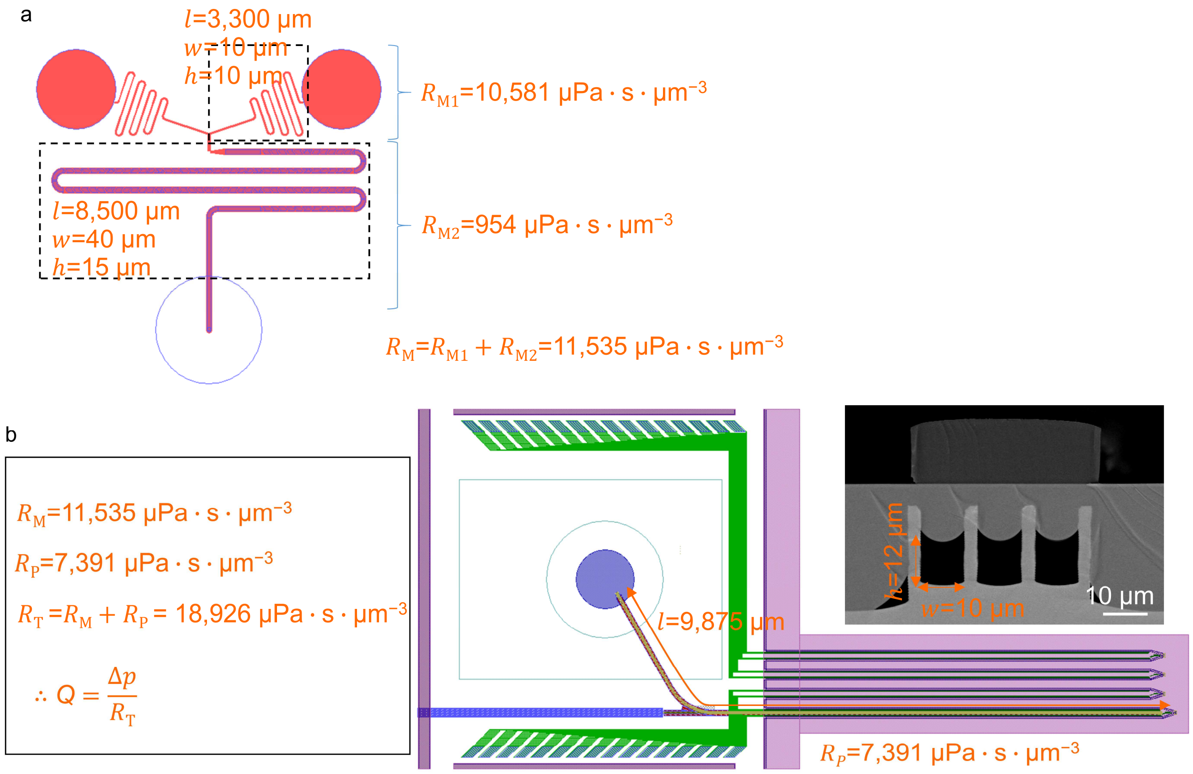

Supplementary Figure 4. Estimation of flow rate and hydraulic resistance: (a) Design of the microfluidic mixer and multifunctional multi-shank MEMS neural probe and calculated fluidic resistances of each part through length (*l*), width (*w*), and height (*h*) of the channels. R_M is fluidic resistance of the microfluidic mixer R_P is fluidic resistance of the probe; (b) Calculated total fluidic resistance for prediction of flow rate R_T is total fluidic resistance.

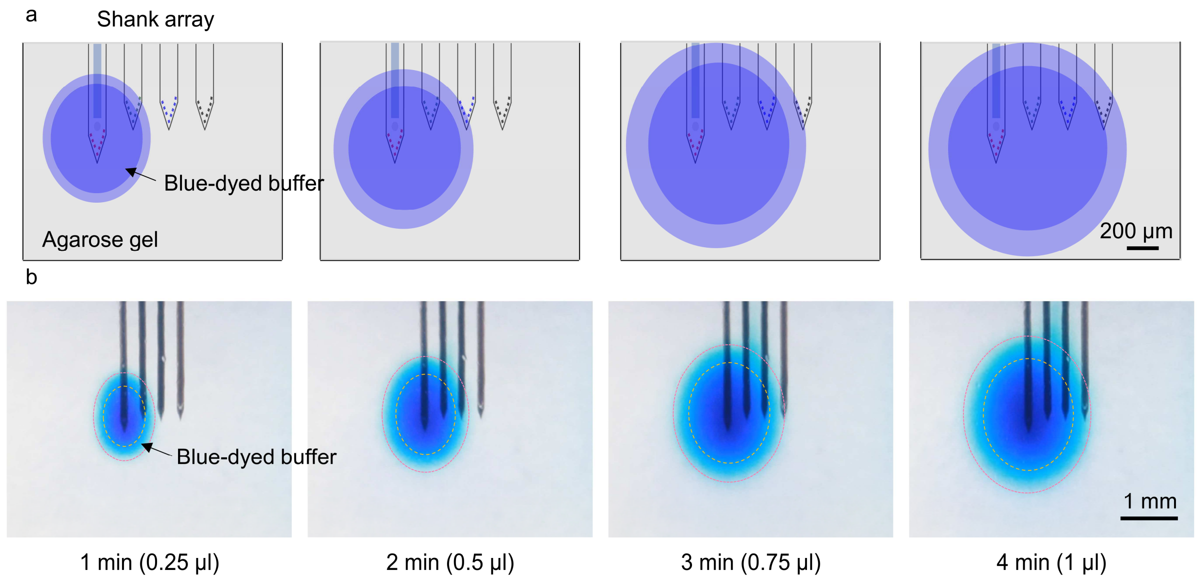

Supplementary Figure 5. Estimation of dispersion profile in brain: (a) Schematic diagram on dispersion of blue-dyed buffer in 0.9% agarose gel; (b) Sequential photographs of dispersed blue-dyed buffer in 0.9% agarose gel when infused using the fabricated multifunctional multi-shank MEMS neural probe when the input pressure and flow rate were 110 kPa and 250 nl·mim^{-1}.

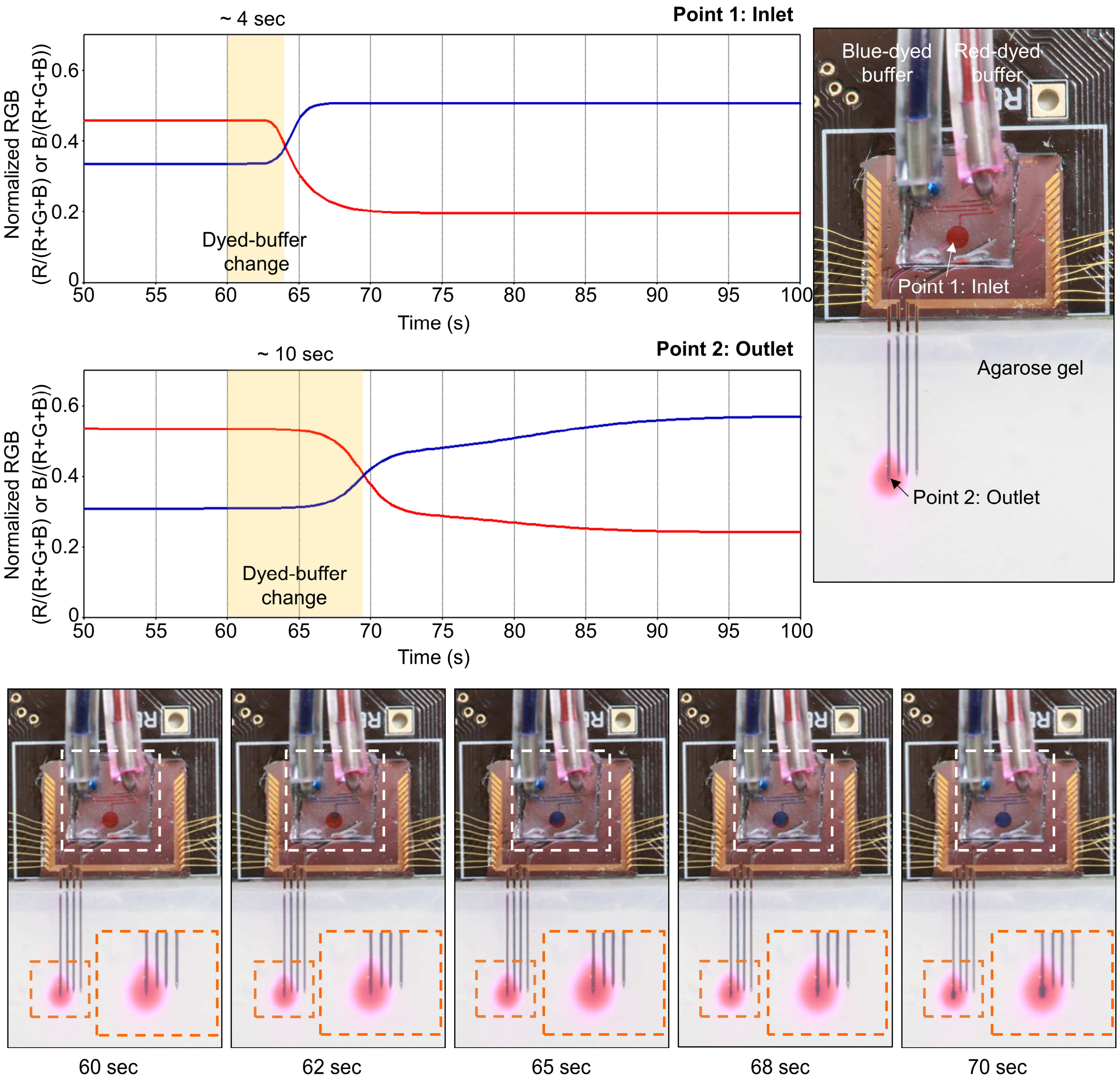

Supplementary Figure 6. Estimation of the swept volume. Red-dyed and blue-dyed buffers were sequentially injected through the mixer and microfluidic channels of the probe in 0.9% agarose gel. Plots of normalized RGB and the optical images show that a sequential process in which the remaining red-dyed buffer completely flushed out after switching to the blue-dyed buffer.

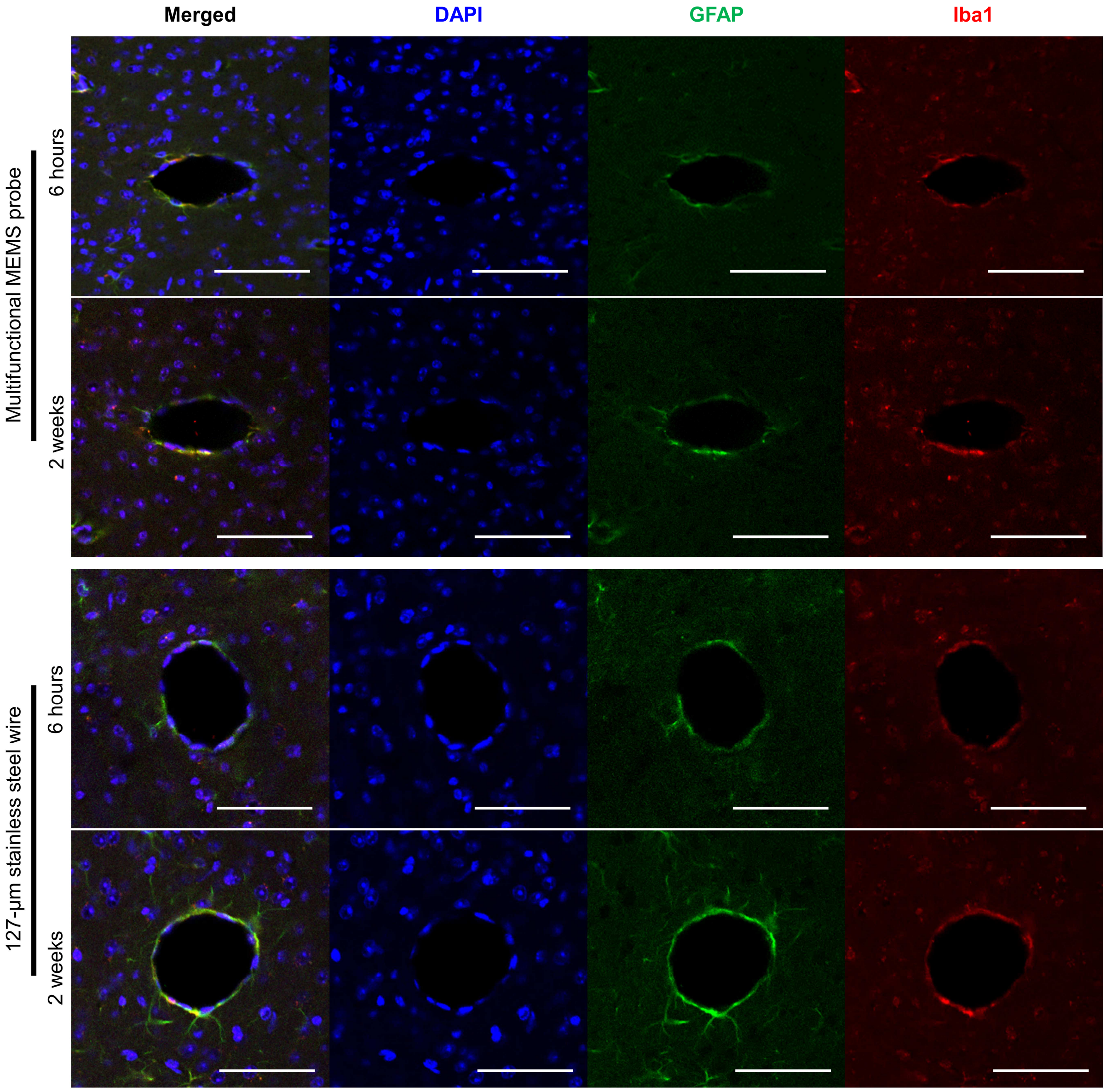

Supplementary Figure 7. Immune responses to implanted multifunctional multi-shank MEMS neural probe and 127 μm stainless steel wire in mouse brain: Representative fluorescent confocal images of the 50 μm horizontal brain slices show immune response 6 hours and 2 weeks after implantation of the probe and wire in the hippocampus of wild-type mouse (scale bars = 100 μm).

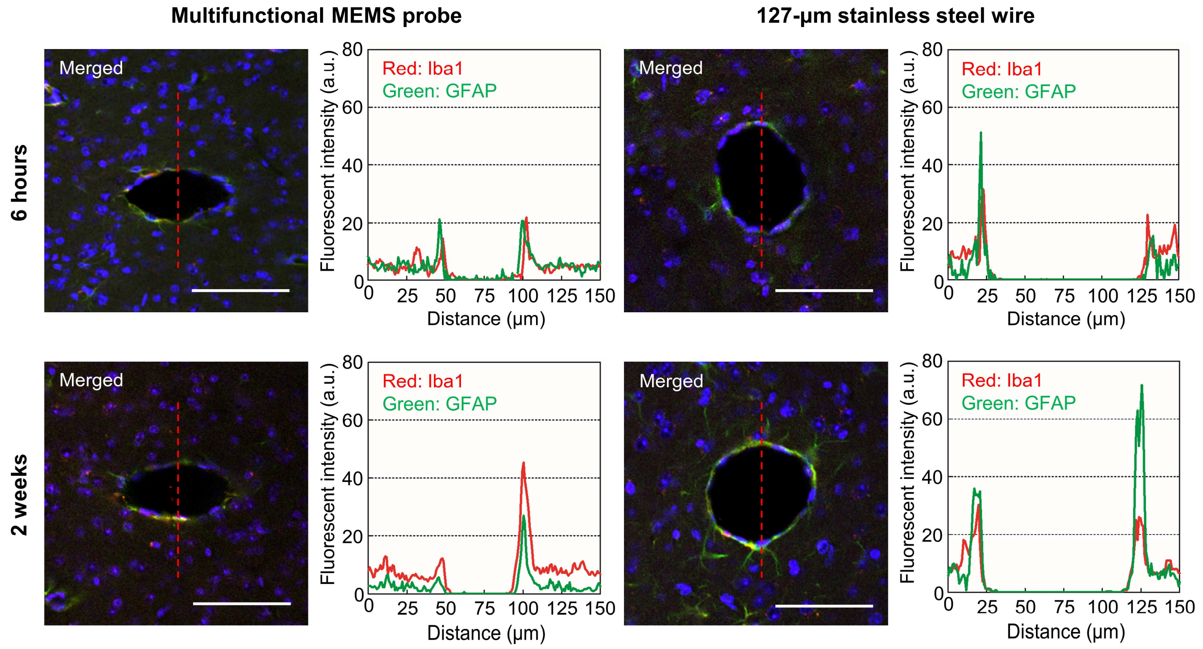

Supplementary Figure 8. Comparison of insertion damage and immune response around multifunctional MEMS probe and 127-μm stainless steel wire: Representative line plots from the red line at the center show less insertion damage and immune responses than 127-μm stainless steel wire due to small thickness of 40 μm of the multifunctional MEMS probe (scale bars = 100 μm).

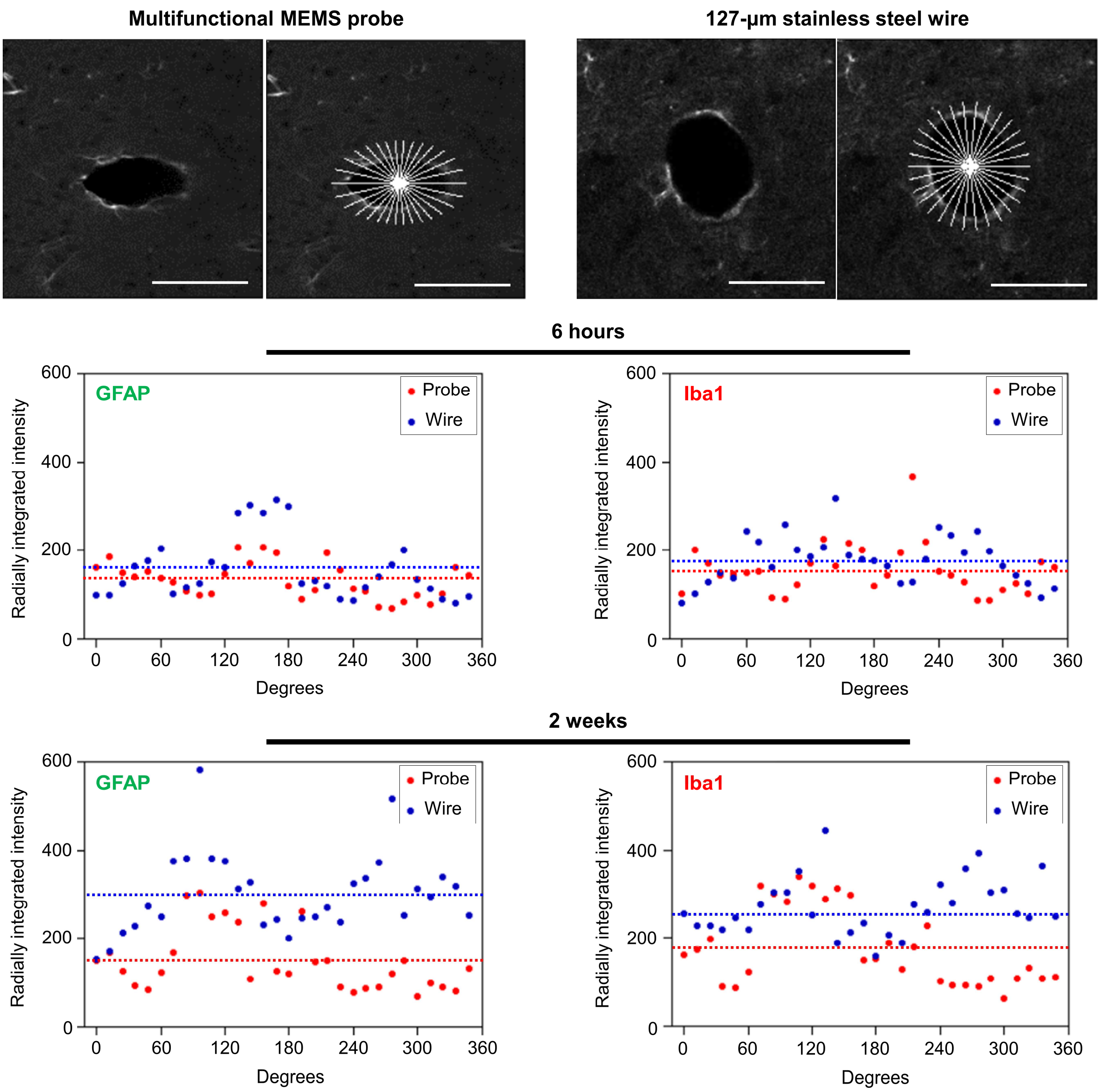

Supplementary Figure 9. Comparison of immune response around multifunctional MEMS probe and 127-μm stainless steel wire: Representative oval profiles from the center to damaged regions show similar or less immune responses than 127-μm stainless steel wire due to small insertion damage of the multifunctional MEMS probe. The lines in the graph represent the average intensity of fluorescence around probe (red line) and wire (blue line) (scale bars = 100 μm).

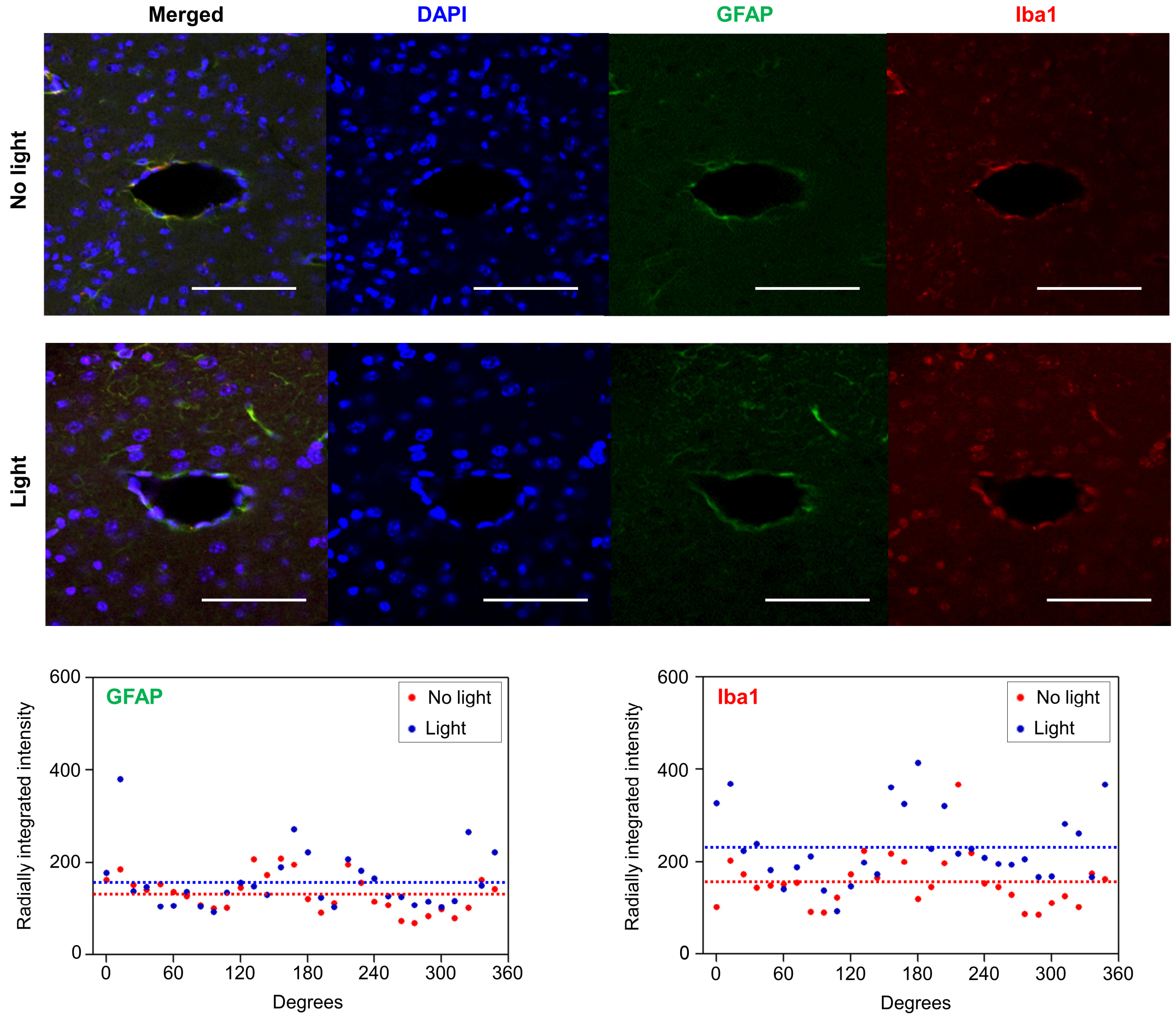

Supplementary Figure 10. Comparison of immune response in the case no optical stimulation and optical stimulation around multifunctional MEMS probe: Representative images and oval profiles show no difference in the immune response around the probe by optical stimulation for short term (1 Hz, 50 % duty cycle, 30 min x 3 times). The lines in the graph represent the average intensity of no light (red line) and light (blue line) (scale bars = 100 μm).

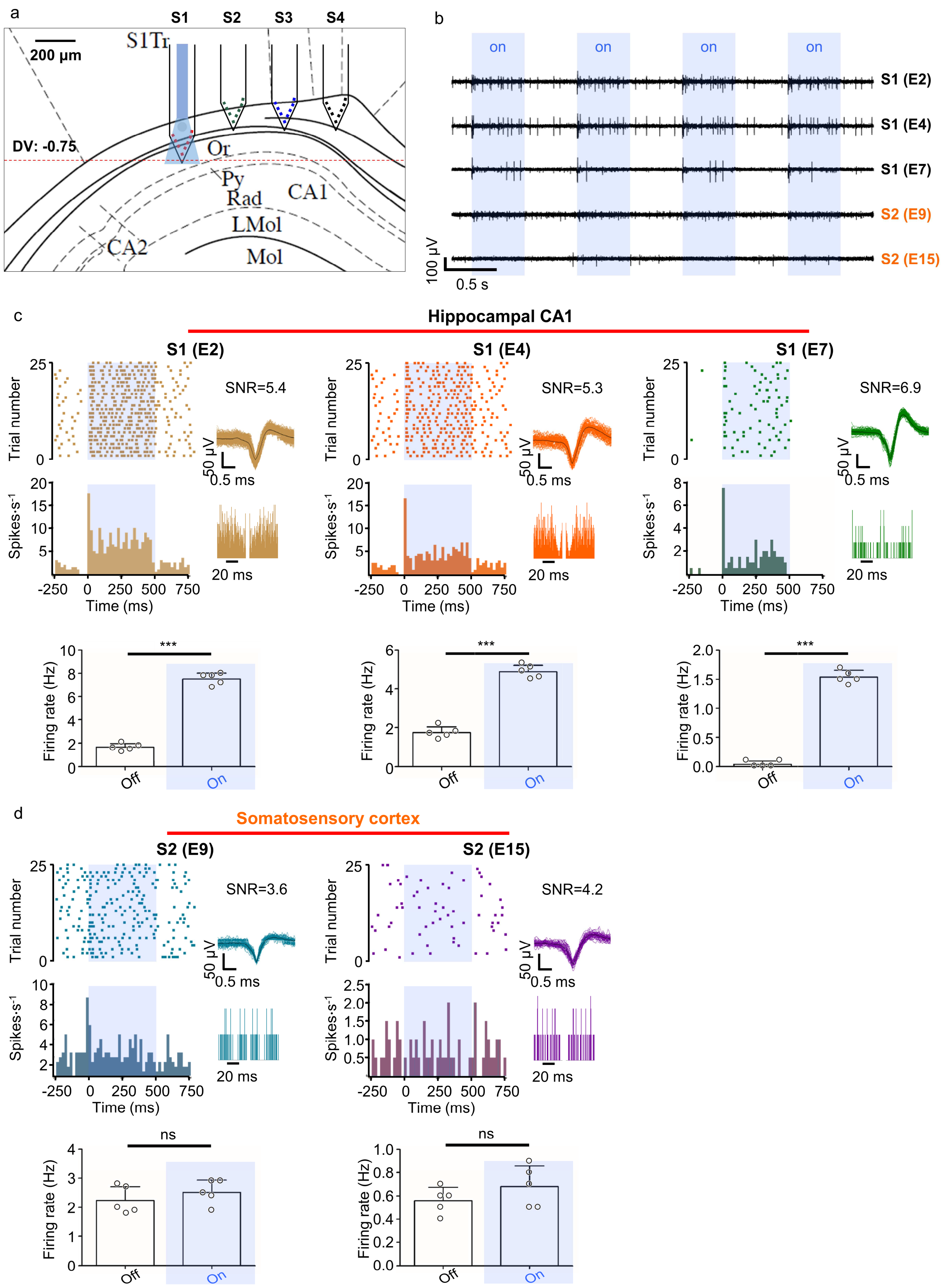

Supplementary Figure 11. In vivo experimental results on localized optical stimulation with the multifunctional multi-shank MEMS probe: (a) The multifunctional multi-shank MEMS neural probe

inserted in hippocampal CA1 (shank 1) and somatosensory cortex (shank 2) of transgenic Thy1-ChR2-YFP mice; (b) Neural signals recorded from the electrodes in shank 1 inserted in hippocampal CA1 and shank 2 inserted in somatosensory cortex during light stimulation (1 Hz, 50% duty cycles; blue background indicates the onset of light); (c) Rater plots of detected neural signals from electrodes in hippocampal CA1 during off and on cycles and peristumulus time histograms (PSTH) of 25 trials and sorted neural spike signals with the autocorrelograms and SNR of the sorted signals and comparison of the firing rate of off and on cycles during optical stimulation show a significant increase in firing rate by directly optical stimulation; (d) Rater plots of detected neural signals from electrodes in somatosensory cortex during off and on cycles and peristumulus time histograms (PSTH) of 25 trials and sorted neural spike signals with the autocorrelograms and SNR of the sorted signals and comparison of the firing rate of off and on cycles during optical stimulation show no difference in firing rate by indirectly optical stimulation. Error bars indicate s.d. (S1(E2): **** $P < 0.0001$, t=22.31, S1(E4): **** $P < 0.0001$, t=15.7, S1(E7): *** $P < 0.0001$, t=26.52, S2(E9): ns $P = 0.3483$, t=0.9962, S2(E15): ns $P = 0.2415$, t=1.265. d.f. = 8 and n = 5 for all, n=firing rate for 5 trials, Two-tailed t-test).

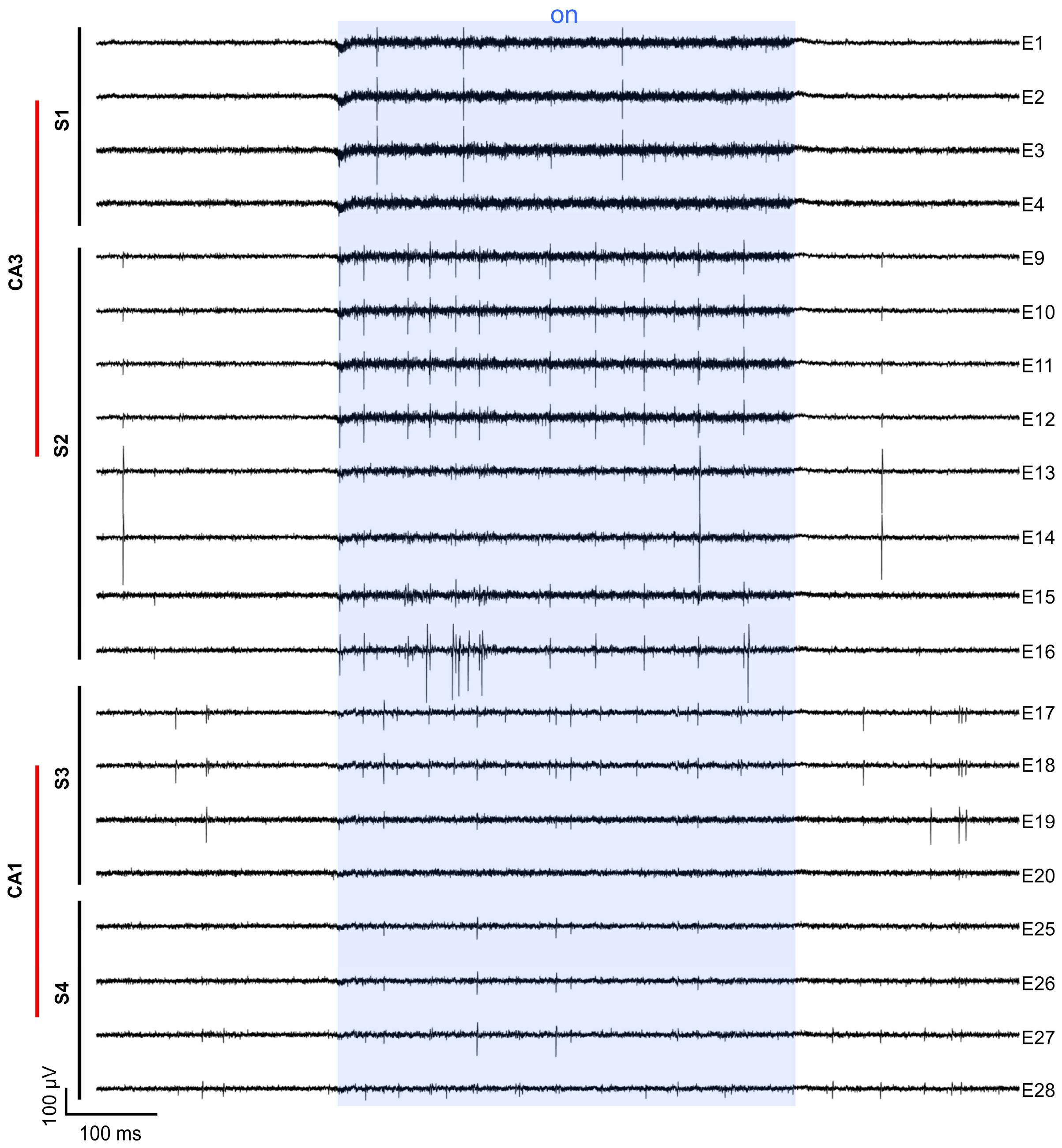

Supplementary Figure 12. Expanded transient plot during light stimulation: Neural signals recorded from over half the electrodes in shank 1, 2 inserted in hippocampal CA3 and shank 3, 4 inserted in hippocampal CA1 during light stimulation (1 Hz, 50 % duty cycles; blue background indicate light onset).

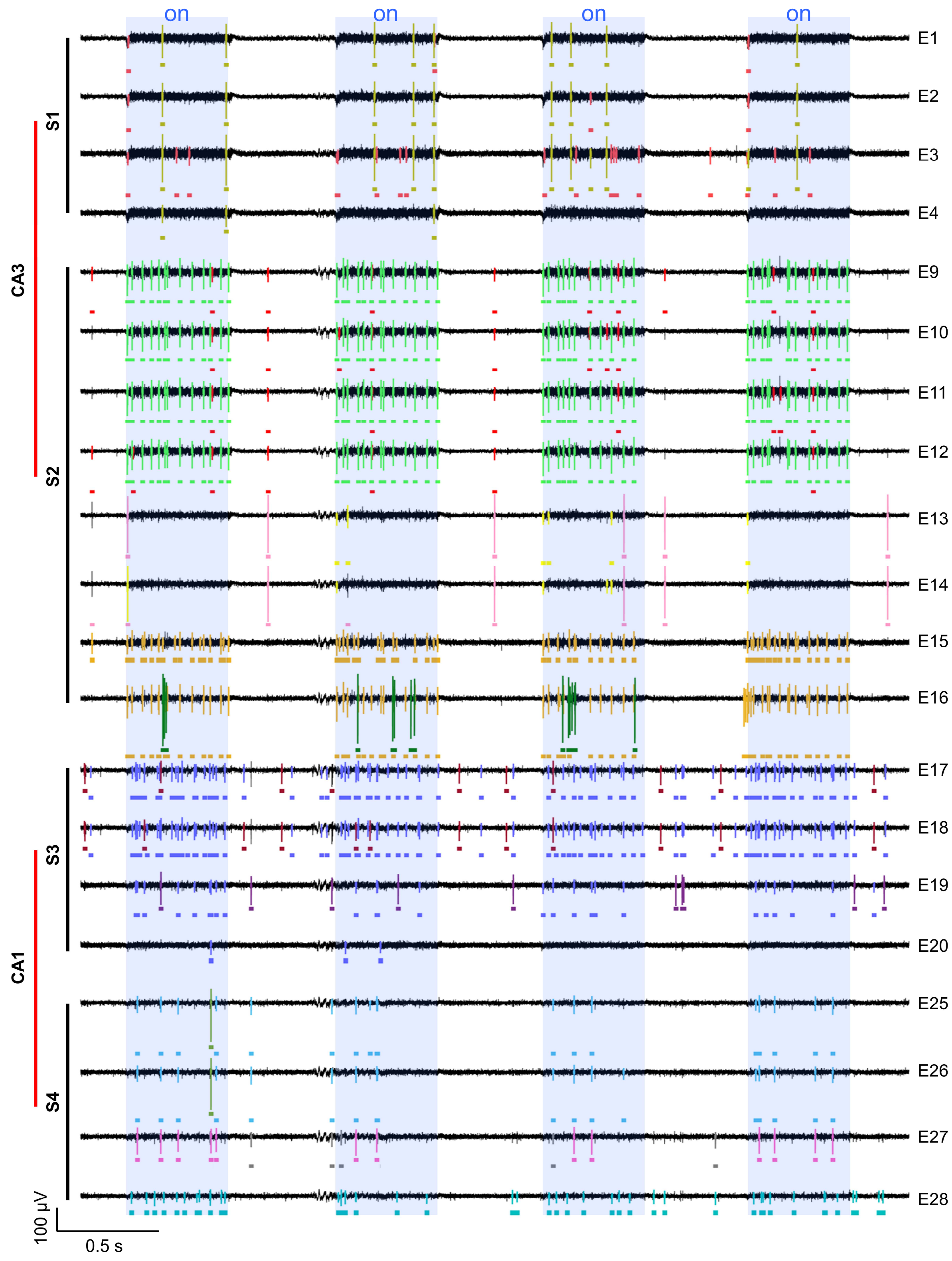

Supplementary Figure 13. Raster plot of all sorted neural signals from spontaneous activities before optical stimulation and light-induced activities during optical stimulation (blue mark indicates light onset).

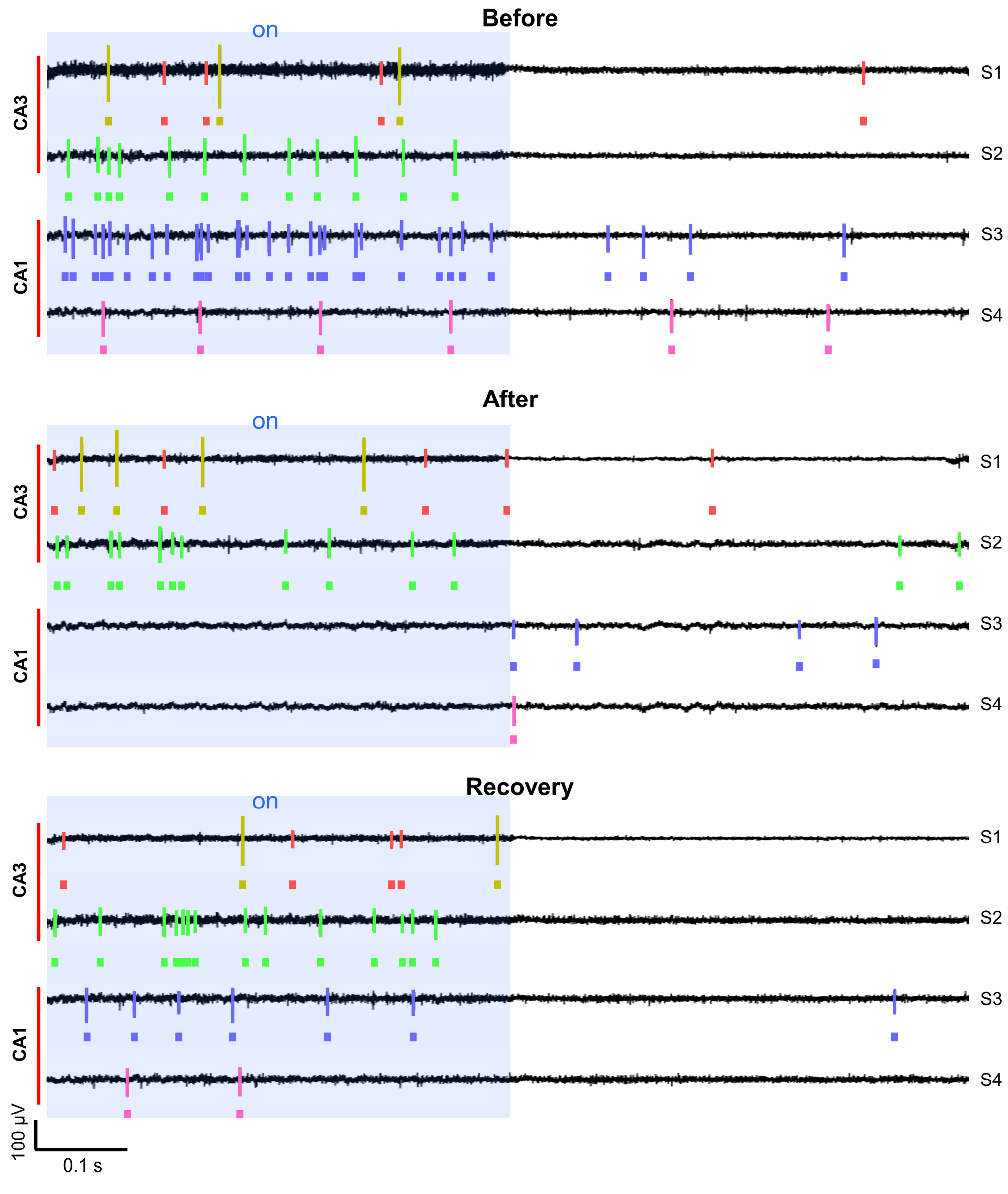

Supplementary Figure 14. Enlarged raster plot of sorted neural signals before and after injection of CNQX and AP5 including the recovery period (blue mark indicates light onset).

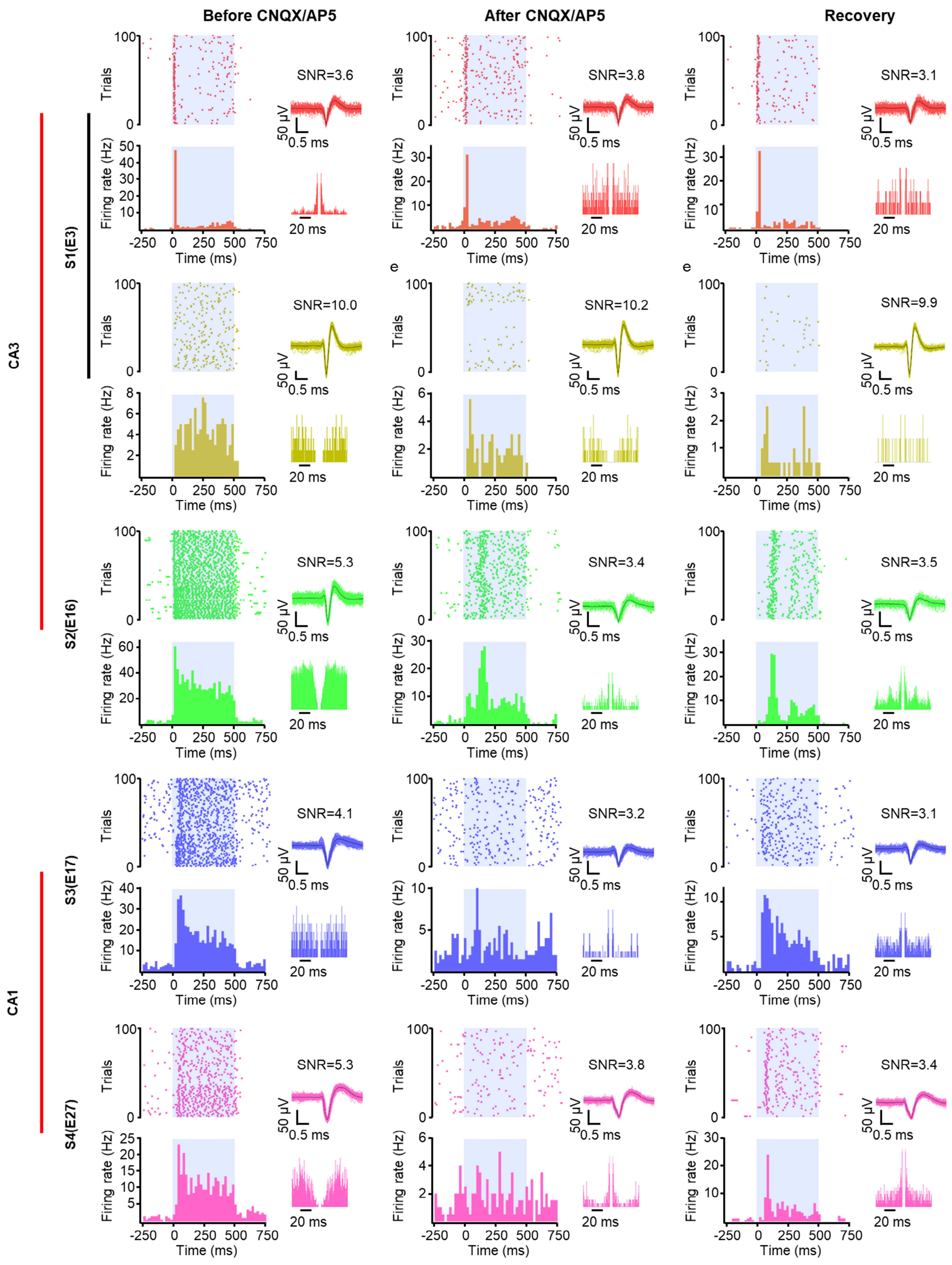

Supplementary Figure 15. Comparison of the amplitude and the firing rate of sorted neural signals before and after injection of CNQX and AP5 and after the recovery: Representative raster plots of detected neural signals from electrodes in hippocampal CA3 and CA1 during off and on cycles

and peristumulus time histograms (PSTH) of 100 trials and sorted neural spike signals with the autocorrelograms and SNR of the sorted signals before and after injection of CNQX/AP5 and after recovery.

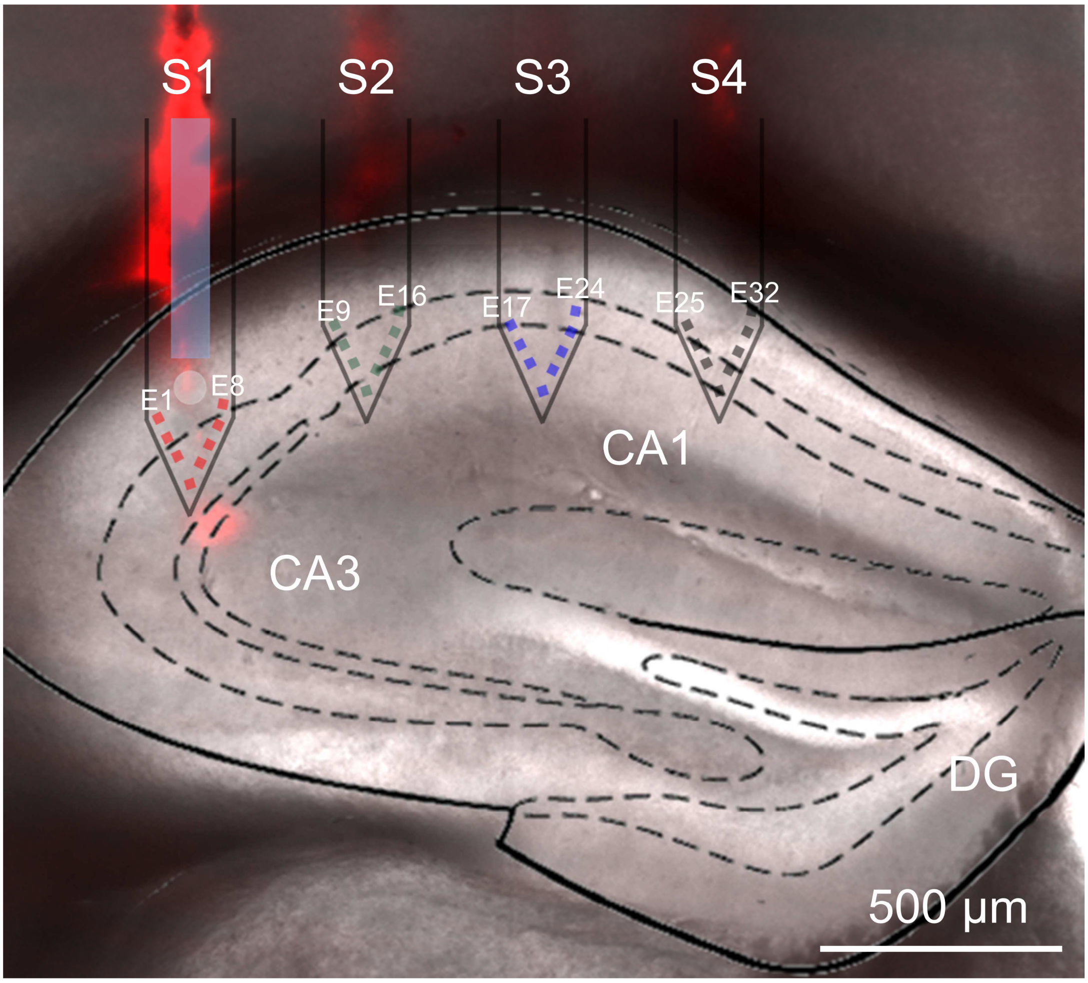

Supplementary Figure 16. Confocal image of the stained brain slice using DiI cell-labelling solution (shown in red): Fluorescent image shows successful insertion of the multifunctional multi-shank MEMS neural probe in target region (hippocampal CA3-CA1).

Supplementary Note 1. Estimation on light intensity inside the brain

To estimate a light intensity transmitted from the SU-8 waveguide inside the brain, we used equations of power-intensity transformation[5-7]. Equations for power-intensity transformation can be expressed as:

$$I = P_\mathrm{s} \cdot \eta_\mathrm{scatter} \cdot \Phi_\mathrm{geometry} \tag{1}$$

In the equation 1, I is intensity (mW·mm^{-2}), P_s is the source power (mW). η_scatter is $1/d$ scattering model for $d < 1$ mm for an approximation to the power attenuation. Φ_geometry is geometric dispersion of light.

$$\eta_\mathrm{scatter} = \frac{1}{S(\lambda)d + 1} \tag{2}$$

In the equation 2, $S(\lambda)$ is mean scattering coefficient at a certain wavelength (S(blue)=7.2). d is the axial distance (mm).

$$\Phi_\mathrm{geometry} = \frac{1}{\pi((r_0 + d\tan(\alpha))^2}$$
$$\alpha = \sin^{-1}\left(\frac{NA_\mathrm{waveguide}}{n_\mathrm{brain}}\right), \qquad NA_\mathrm{waveguide} = \sqrt{n_\mathrm{core}^2 - n_\mathrm{cladding}^2} \tag{3}$$

In the equation 3, $NA_\mathrm{waveguide}$ is the numerical aperture of the waveguide. n is refractive index ($n_\mathrm{core} = 1.54$, $n_\mathrm{cladding} = 1.46$, and $n_\mathrm{brain} = 1.36$). r_0 is equivalent radius (r_0=13.82 μm). Based above equations, we calculated the light intensity along the axial distance from the end of the SU-8 waveguide in brain.

Supplementary Note 2. Estimation of the flow rate and hydraulic resistance

To estimate hydraulic resistance from the microfluidic channels and the flow rate under input pressure, we used the below equations[8]. Equations for calculation of hydraulic resistance and flow rate can be expressed as:

$$Q = \frac{\Delta p}{R} \cdot \frac{6}{10^5} \tag{4}$$

In the equation 4, Q is flow rate (nl·min^{-1}), Δp is input pressure (Pa), R is fluidic resistance.

$$R = n \cdot \frac{1}{2\eta} \cdot \frac{A^3}{lP^2}$$
$$A = h \cdot w \tag{5}$$
$$P = 2h + 2w$$

In the equation 5, n is the number of fluidic channels, η is dynamic viscosity (Pa · s), A, l, and P are the cross-sectional area (μm^3), length (μm), and cross-sectional perimeter (μm) of the microfluidic cannel. h is the height of microfluidic channel, w is the width of microfluidic channel.

Based above equations, we calculated the hydraulic resistance of microfluidic channel and estimated the flow rate under input pressure.

Supplementary References

1. Park, S., *et al.* One-step optogenetics with multifunctional flexible polymer fibers. *Nat Neurosci.* **20**, 612-619 (2017).
2. Jeong, J.W., *et al.* Wireless Optofluidic Systems for Programmable In Vivo Pharmacology and Optogenetics. *Cell* **162**, 662-674 (2015).
3. Rubehn, B., Wolff, S.B., Tovote, P., Luthi, A. & Stieglitz, T. A polymer-based neural microimplant for optogenetic applications: design and first in vivo study. *Lab Chip* **13**, 579-588 (2013).
4. Fang, Q., *et al.* Monte Carlo simulation of photon migration in 3D turbid media accelerated by graphics processing units. *Opt. Express* **17**, 20178-20190 https://doi.org/10.1364/OE.17.020178 (2009).
5. Son, Y., *et al.* In vivo optical modulation of neural signals using monolithically integrated two-dimensional neural probe arrays. *Sci Rep* **5**, 15466 https://doi.org/10.1038/srep15466 (2015).
6. Stark, E., *et al.* G. Diode probes for spatiotemporal optical control of multiple neurons in freely moving animals. *J Neurophysiol* **108**, 349-363 (2012).
7. Aravanis, A.M., *et al.* An optical neural interface: in vivo control of rodent motor cortex with integrated fiberoptic and optogenetic technology. *J Neural Eng* **4**, S143-156 (2007).
8. Lee, H.J., *et al.* A multichannel neural probe with embedded microfluidic channels for simultaneous in vivo neural recording and drug delivery. *Lab Chip* **15**, 1590-15697 (2015).
