# Supplementary material for: Multifunctional multi-shank neural probe for investigating and modulating long-range neural circuits in vivo
Source: Nat Commun. 2019 Aug 22;10:3777. doi: 10.1038/s41467-019-11628-5 (PMC6706395; doi:10.1038/s41467-019-11628-5)
Supplement: Supplementary file 2 — Description of Additional Supplementary Files [file 41467_2019_11628_MOESM2_ESM.pdf]

Description of Additional Supplementary Files

File name: Supplementary Code 1

Description: The customized Matlab code for sorting spikes from the neural signal recorded with microelectrode array of the multifunctional neural probe.
