# Supplementary material for: Multifunctional multi-shank neural probe for investigating and modulating long-range neural circuits in vivo
Source: Nat Commun. 2019 Aug 22;10:3777. doi: 10.1038/s41467-019-11628-5 (PMC6706395; doi:10.1038/s41467-019-11628-5)
Supplement: Supplementary file 4 — Reporting Summary [file 41467_2019_11628_MOESM4_ESM.pdf]

nature research

Corresponding author(s): DBPR, NCOMMS-19-01625A

Last updated by author(s): Jul 8, 2019

Reporting Summary

Nature Research wishes to improve the reproducibility of the work that we publish. This form provides structure for consistency and transparency in reporting. For further information on Nature Research policies, see Authors & Referees and the Editorial Policy Checklist.

Statistics

For all statistical analyses, confirm that the following items are present in the figure legend, table legend, main text, or Methods section.

n/a	Confirmed	
☐	☒	The exact sample size (*n*) for each experimental group/condition, given as a discrete number and unit of measurement
☐	☒	A statement on whether measurements were taken from distinct samples or whether the same sample was measured repeatedly
☐	☒	The statistical test(s) used AND whether they are one- or two-sided<br>*Only common tests should be described solely by name; describe more complex techniques in the Methods section.*
☒	☐	A description of all covariates tested
☒	☐	A description of any assumptions or corrections, such as tests of normality and adjustment for multiple comparisons
☐	☒	A full description of the statistical parameters including central tendency (e.g. means) or other basic estimates (e.g. regression coefficient) AND variation (e.g. standard deviation) or associated estimates of uncertainty (e.g. confidence intervals)
☐	☒	For null hypothesis testing, the test statistic (e.g. *F*, *t*, *r*) with confidence intervals, effect sizes, degrees of freedom and *P* value noted<br>*Give P values as exact values whenever suitable.*
☒	☐	For Bayesian analysis, information on the choice of priors and Markov chain Monte Carlo settings
☐	☒	For hierarchical and complex designs, identification of the appropriate level for tests and full reporting of outcomes
☒	☐	Estimates of effect sizes (e.g. Cohen's *d*, Pearson's *r*), indicating how they were calculated

Our web collection on statistics for biologists contains articles on many of the points above.

Software and code

Policy information about availability of computer code

Data collection	NanoZ 1.4 (Neuralynx), Cheetah 5.6 (Neuralynx), Matlab2016a (Matlab), ZEN microscope software (Zeiss)
Data analysis	Matlab2016a (Matlab), GraphPad Prism 7.04 (GraphPad Prism)<br>Signal analysis was performed on Matlab. And, customized Matlab code are included in supplementary information. Statistical analysis was performed on GraphPad Prism.

For manuscripts utilizing custom algorithms or software that are central to the research but not yet described in published literature, software must be made available to editors/reviewers. We strongly encourage code deposition in a community repository (e.g. GitHub). See the Nature Research guidelines for submitting code & software for further information.

Data

Policy information about availability of data

All manuscripts must include a data availability statement. This statement should provide the following information, where applicable:

- Accession codes, unique identifiers, or web links for publicly available datasets
- A list of figures that have associated raw data
- A description of any restrictions on data availability

All relevant data are included in the main manuscript and the supplementary Information. All data including additional data are available from the corresponding author upon request.

Field-specific reporting

Please select the one below that is the best fit for your research. If you are not sure, read the appropriate sections before making your selection.

☒ Life sciences ☐ Behavioural & social sciences ☐ Ecological, evolutionary & environmental sciences

For a reference copy of the document with all sections, see nature.com/documents/nr-reporting-summary-flat.pdf

Life sciences study design

All studies must disclose on these points even when the disclosure is negative.

Sample size	We did not use a computational method to determine sample size. We determined sample size to be appropriate based on the consistency and magnitude of measurable differences in experiments.
Data exclusions	No data were excluded.
Replication	Data was collated in multiple experiments. No experiments failed to replicate.
Randomization	Mice were randomly assigned to experiments.
Blinding	The investigators were not blinded to group allocation during data collection. However, all data analysis was performed automatically using MATLAB, with the same code run on each experiments.

Reporting for specific materials, systems and methods

We require information from authors about some types of materials, experimental systems and methods used in many studies. Here, indicate whether each material, system or method listed is relevant to your study. If you are not sure if a list item applies to your research, read the appropriate section before selecting a response.

Materials & experimental systems

n/a	Involved in the study	
☐	☒	Antibodies
☒	☐	Eukaryotic cell lines
☒	☐	Palaeontology
☐	☒	Animals and other organisms
☒	☐	Human research participants
☒	☐	Clinical data

Methods

n/a	Involved in the study	
☒	☐	ChIP-seq
☒	☐	Flow cytometry
☒	☐	MRI-based neuroimaging

Antibodies

Antibodies used	We used anti-GFAP antibody (abcam ,ab10062, GF5, 1:500), anti-Iba1 antibody (abcam, ab48004, polyclonal, 1:200), goat anti-mouse conjugated Alexa Fluor 488 (abcam, ab150113, polyclonal, 1:500), donkey anti-goat conjugated Alexa Fluor 594 (abcam, ab150136, polyclonal, 1:200)
Validation	Validation is provided on the manufacturer`s website for each product.

Animals and other organisms

Policy information about studies involving animals; ARRIVE guidelines recommended for reporting animal research

Laboratory animals	Adult male mice (C57BL/6J, 10 weeks) and adult male transgenic mice (C57BL/6J, Thy1-ChR2-YFP, 10 weeks) were used in the study.
Wild animals	NA
Field-collected samples	NA
Ethics oversight	All of the procedures that involved the use of mice were approved by the Korea Institute of Science and Technology (KIST) in Seoul, Korea, and the procedures were conducted in accordance with the ethical standards stated in the Animal Care and Use Guidelines of KIST.

Note that full information on the approval of the study protocol must also be provided in the manuscript.
